# Sociodemographic differences in motives for food selection: results from the LoCard cross-sectional survey

**DOI:** 10.1186/s12966-021-01139-2

**Published:** 2021-06-02

**Authors:** Hanna Konttinen, Otto Halmesvaara, Mikael Fogelholm, Hannu Saarijärvi, Jaakko Nevalainen, Maijaliisa Erkkola

**Affiliations:** 1 Faculty of Social Sciences, University of Helsinki, P.O. Box 18, Helsinki, 00014 Finland; 2 Department of Food and Nutrition, University of Helsinki, P.O. Box 66, Helsinki, 00014 Finland; 3 Faculty of Management and Business, Tampere University, Tampere, Finland; 4 Faculty of Social Sciences (Health Sciences), Tampere University, Tampere, Finland

**Keywords:** Socioeconomic status, Age, Gender, Disparities, Motives, Eating behaviour, Food choice questionnaire, Vegetarian diet, Price

## Abstract

**Background:**

Although sociodemographic differences in dietary intake have been widely studied, the up-to-date evidence on the corresponding variations in motives for food selection is limited. We investigated how sociodemographic characteristics and special diets in households are associated with the relative importance of various food motives.

**Methods:**

Participants were members of the S Group loyalty card program across Finland who consented to release their grocery purchase data to be used for research purposes and responded to a web-based questionnaire in 2018 (LoCard study). Self-reported information on sociodemographic factors (age, gender, marital status, living situation, education, household income), special diets in household and food motives (Food Choice Questionnaire) were utilized in the present analyses (*N* = 10,795). Age- and gender-adjusted linear models were performed separately for each sociodemographic predictor and motive dimension (derived by factor analysis) outcome. The importance of each sociodemographic predictor was evaluated based on an increase in *R*^2^ value after adding the predictor to the age- and gender-adjusted model.

**Results:**

Age emerged as a central determinant of food motives with the following strongest associations: young adults emphasized convenience (∆*R*^2^ = 0.09, *P* < 0.001) and mood control (∆*R*^2^ = 0.05, *P* < 0.001) motives more than middle-aged and older adults. The relative importance of cheapness decreased with increasing socioeconomic position (SEP) (∆*R*^2^ = 0.08, *P* < 0.001 for income and ∆*R*^2^ = 0.04, *P* < 0.001 for education). However, the price item (“is good value for money”) depicting the concept of worth did not distinguish between SEP categories. Considerations related to familiarity of food were more salient to men (∆*R*^2^ = 0.02, *P* < 0.001) and those with lower SEP (∆*R*^2^ = 0.03, *P* < 0.001 for education and ∆*R*^2^ = 0.01, *P* < 0.001 for income). Respondents living in households with a vegetarian, red-meat-free, gluten-free or other type of special diet rated ethical concern as relatively more important than households with no special diets (∆*R*^2^ = 0.02, *P* < 0.001).

**Conclusions:**

We observed sociodemographic differences in a range of food motives that might act as barriers or drivers for adopting diets that benefit human and planetary health. Interventions aiming to narrow SEP and gender disparities in dietary intake should employ strategies that take into account higher priority of familiarity and price in daily food selection in lower-SEP individuals and males.

**Supplementary Information:**

The online version contains supplementary material available at 10.1186/s12966-021-01139-2.

## Background

There are substantial differences in the nutritional quality of diets between various sociodemographic groups, which are likely to contribute to health inequalities [1]. In high-income countries, women and individuals with higher socioeconomic position (SEP) tend to consume more vegetables and less red meat than men and those with lower SEP [2–5]. While achieving equity in health is a priority in many initiatives and agendas [6], there is simultaneously an urgent need for a radical transformation of the current western diets to improve human health and environmental sustainability. The EAT-Lancet commission of leading scientists recently argued that by 2050 the global consumption of fruits, vegetables, nuts, and legumes will have to double, and the consumption of red meat will have to reduce more than 50% [7]. This sizeable shift in diets is not possible without multiple actions at various societal levels of affluent countries. To ensure the acceptability and effectiveness of these actions across sociodemographic groups [8], enhanced knowledge on motives underlying food selection in different population segments is needed. People differ in the importance they place on various health- and non-health-related motives in their daily food selection (e.g. taste, health, price, convenience, natural content, familiarity, ethical concern), and these differences are related to variations in dietary intake [9–13]. While motives, such as health, natural content or ethical concern, have been linked with healthier dietary intake (e.g. more vegetables and less meat products), the opposite has been observed for price, convenience or familiarity [10, 12, 13]. Therefore, obtaining data on the current sociodemographic patterns in food motives would aid in designing more efficient and tailored food-related interventions as well as identifying consumer segments for food product innovation and marketing.

However, although sociodemographic variations in diets have been widely studied, the corresponding information on food motives is scarcer. There is some evidence that women rate most motives as more important than men, especially those related to health and weight control [9, 11, 12, 14, 15]. Compared to younger people, older individuals may value more “long-term-oriented” motives, including health, natural content and ethical concern [9, 11, 15]. Scattered data further imply that lower education and/or income associates with placing higher importance on price and familiarity motives and lower importance on health and weight control motives [12, 16–20]. It is though noteworthy that many of these previous studies have been conducted 10–20 years ago. As public attitudes to and interest in various specific diets and food ingredients (e.g. veganism, low-carbohydrate diets, gluten-free diet, food additives) are constantly changing [21], obtaining up-to-date information on food motives is essential. Moreover, in the light of the need for a considerable reduction in the global consumption of red meat, the key motives that differentiate between people who follow a more or a less meat-based diet should be identified.

When studying motives for food selection, an important issue to take into account is that people usually consider several motives as personally relevant. Consequently, conflicts between motives (e.g. price, convenience or taste vs. health) are common in specific food selection situations, making it necessary for individuals to prioritize them [22–24]. It has been proposed that besides examining the absolute importance of single motives, the relative importance should also be analysed (as derived by dividing individuals’ scores on a single motive by their mean rating across all the motives) [12]. Analysing individuals’ motive priorities may better reflect the complexity of the motive structure in that relatively unimportant motives might not affect food selection, even though their absolute importance is high [25]. Nonetheless, data on sociodemographic differences in the relative importance of various motives is limited.

The present study aimed to extend earlier literature by using recent data of more than 10,000 Finnish adults to investigate how sociodemographic characteristics and special diets in households are related to the relative importance of health, sensory appeal, mood control, convenience, natural content, price, weight control, familiarity and ethical concern motives.

## Methods

### Participants and study design

The questionnaire data collected in June–August 2018 in the Loyalty Card (LoCard) study were utilized in the present research. The data (pseudonymised) were derived from the S Group, which is the largest commercial operator in grocery retail sector in Finland. Full details of the data collection process (with a participant flow chart) have been reported by Vuorinen et al. [26]. Approximately 1.1 million members of the S Group loyalty card program across Finland received an email asking for permission to use their loyalty card data (i.e. data concerning their grocery purchases) for research purposes. The email also included a link, which randomly assigned the members to voluntarily complete one of the three possible questionnaires (all containing questions on sociodemographic factors), one of which contained questions on food motives. Members who did not have an email address in the retailer’s database, had prohibited the retailer to contact them with any marketing or research-related material, or were under 18 years of age were excluded and did not receive the email. All emails were sent by the S Group who had the loyalty card holders’ contact information. The authors did not possess information on the number of valid email addresses or the proportion of emails reaching the recipients (e.g. bypassing trash email filters). As a reward for taking part, twenty S Group gift cards (each worth of 50 euros) were drawn among those who gave their consent to participate in the study.

In total, 47,066 members consented to give their loyalty card data for the research, and 36,621 (78% of those who consented) responded to the questionnaire of which 12,334 filled the questionnaire including food motive items. The sample analysed in this study consisted of 10,795 participants who answered all 28 items assessing food motives (excluding 1539 participants with missing data on one or more motive items). Based on Cramer’s V statistics [27], there were negligible differences between individuals with and without missing motive item data in terms of gender (χ^2^(1) = 31.7, *p* < 0.001, V = 0.05), marital status (χ^2^(2) = 30.6, *p* < 0.001, V = 0.05), living situation (χ^2^(2) = 62.0, *p* < 0.001, V = 0.07), education (χ^2^(3) = 28.6, *p* < 0.001, V = 0.05), and household income (χ^2^(3) = 20.2, *p* < 0.001, V = 0.04). In terms of age (χ^2^(3) = 306.6, *p* < 0.001, V = 0.16), a weak association was detected with the proportion of 18–29-years-olds being lower (7% vs. 15%) and 65–94-year-olds being higher (29% vs. 14%) in those with missing data.

### Measures

#### Predictors

Sociodemographic characteristics (including age, gender, marital status, living situation, education, household income) were assessed via self-report. Age was categorized into four groups (18–29, 30–44, 45–64, 65–94), while three categories were used to define participants’ marital status (married/cohabiting, divorced/widowed, never married) and living situation (alone, two or more adults, adult [s] and child [ren]). Participants reported their education on a four-point scale (basic, middle, lower academic [i.e., bachelor’s degree], upper academic [i.e., master’s degree]) and gross household income per month on a seven-point scale ranging from less than 1500€ to 9000€ or more. Those who indicated that they do not know their household income or do not want to answer (*N* = 685) were excluded from the models containing household income variable. Household income was divided by the weighted sum of the number of household adult and child members: a weight of 1.0 was given for the first adult of the household, 0.7 for all other adults, and 0.5 for children under the age of 18 [28]. This scaled household income variable was then divided into quartiles (1st quartile ranging from 127.1€ to 1458.3€, 2nd quartile from 1458.4€ to 2250.0€, 3rd quartile from 2250.1€ to 3088.2€, 4th quartile from 3088.3€ to 9750.0€).

Respondents were asked to indicate whether members of their household had any special diets. They were able to choose one or more diets and the following categories were utilized in the present analyses: 1) no special diet; 2) lactose-free diet (excl. no red meat, vegetarian, gluten-free); 3) gluten-free diet (excl. no red meat, vegetarian); 4) diet that does not include red meat; 5) vegetarian and/or vegan diet (excl. no red meat); 6) other diets (incl. food allergies). The rationale for this categorization was that we were mainly interested in red-meat-free and vegetarian diets (i.e. special diets that are chosen, not followed due to disease or intolerance) in this study.

#### Outcomes

Food motives were assessed with a 28-item shortened version of the FCQ [9, 12]. The original FCQ was developed by using a demographically heterogeneous sample from the UK and it contains 36 items measuring nine different motivational dimensions (health, mood control, sensory appeal, convenience, natural content, price, weight control, familiarity, ethical concern). Respondents are asked to rate the statement “It is important to me that the food I eat on a typical day …” for each item on a four-point scale (from “1=not at all important” to “4 = very important”). A previous Finnish research applied a shortened version of the FCQ in which 13 items were excluded and three items tapping ethical/political aspects of food purchasing were added [12]. In the present study, we use the same version with the exception that one familiarity item (“is familiar”) and one price item (“is good value for money”) was added to ensure that there were at least two items per each dimension.

The structure of the 28-item FCQ in this sample was examined using exploratory factor analyses. The results from the nine-factor model indicated that most motive dimensions were rather well-replicated (for details, see Additional file 1). The three mood items did not load on the same factor (e.g. “keeps me awake/alert” had the strongest loading on the health factor and “makes me feel good” on the sensory appeal factor), but to retain comparison with previous research, we combined them to the same dimension in subsequent analyses. The two price items (“is cheap”, “is good value for money”) correlated relatively weakly with each other (*r* = 0.22) which was also reflected in their factor loadings. In this case, we decided to analyse them separately because the former one reflects more the influence of monetary resources, while the latter one reflects more the concept of worth [22].

We derived the absolute importance of health, sensory appeal, mood control, convenience, natural content, weight control, familiarity and ethical concern motives by computing the mean score of the items belonging to the respective dimension. Respondent’s rating of each price item reflected its absolute importance. We then calculated the relative importance of each motive by dividing respondent’s absolute rating of it by his/her mean score on all 28 motive items (see also [12]). This means that scores > 1 for each relative motive indicate that the motive is rated more important than on average all the motive items. As discussed in the introduction, our main focus was on the relative importance of motives, while estimates for the absolute motives are reported in Additional file 2.

### Statistical methods

The data were analysed using IBM SPSS Statistics for Windows, version 25 (IBM Corp., Armonk, N.Y., USA). Multiple linear models were conducted with a motive dimension as the outcome and a sociodemographic factor as the predictor. Each predictor was adjusted for the effects of age and gender (excluding, when age/gender was used as a predictor, then age was adjusted for gender and gender for age). Two types of coding for age was employed: when age was used as a control variable, continuous coding was utilized (age in years), and when age was used as a predictor, it was utilized as a factor (age groups) to detect potential nonlinear associations. As the large size of the dataset tended to result in high statistical significance, the practical importance of each sociodemographic predictor was evaluated based on an increase in *R*^2^ value after adding the predictor to the age- and gender-adjusted model. ∆*R*^2^ values lower than .01 were considered to indicate a predictive value that is practically insignificant. A heatmap was also created to visualize the variation in the strength of the associations between sociodemographic factors and food motive dimensions by using information on these ∆*R*^2^ estimates. In addition to (adjusted) regression coefficients, unadjusted mean and standard deviation values for each motive dimension by sociodemographic groups were calculated. The analyses were conducted using all available data; therefore, the number of participants varied between the models (for details, see Table 1).

**Table 1:** Descriptive characteristics of the LoCard study participants and mean values for relative food motives by sociodemographic groups

		HE^a^	WC	SA	MO	NC	EC	CO	FA	PC	PV
	%	Mean (SD)	Mean (SD)	Mean (SD)	Mean (SD)	Mean (SD)	Mean (SD)	Mean (SD)	Mean (SD)	Mean (SD)	Mean (SD)
**Gender (*N*** **= 10,786)**^**b**^											
Women	66.9	1.024 (0.107)	0.895 (0.177)	1.123 (0.151)	1.017 (0.147)	0.986 (0.201)	0.936 (0.147)	1.057 (0.187)	0.836 (0.217)	1.053 (0.233)	1.217 (0.178)
Men	33.1	1.021 (0.112)	0.907 (0.191)	1.130 (0.178)	0.995 (0.166)	0.958 (0.226)	0.920 (0.163)	1.042 (0.207)	0.895 (0.226)	1.101 (0.267)	1.271 (0.227)
***R***^**2c**^		<.001*	.001**	<.001*	.004***	.004***	.002**	.001***	.015***	.008***	.016***
**Age group (*N*** **= 10,786)**											
18–29	15.0	1.016 (0.122)	0.857 (0.194)	1.131 (0.171)	1.071 (0.144)	0.903 (0.221)	0.880 (0.158)	1.123 (0.192)	0.883 (0.219)	1.141 (0.230)	1.264 (0.197)
30–44	33.1	1.016 (0.114)	0.863 (0.185)	1.137 (0.163)	1.027 (0.149)	0.956 (0.215)	0.912 (0.159)	1.103 (0.188)	0.865 (0.224)	1.083 (0.247)	1.246 (0.203)
45–64	38.0	1.024 (0.107)	0.928 (0.172)	1.129 (0.157)	0.994 (0.154)	0.999 (0.197)	0.944 (0.144)	1.019 (0.185)	0.838 (0.221)	1.047 (0.247)	1.228 (0.193)
65–94	13.9	1.055 (0.103)	0.950 (0.182)	1.083 (0.148)	0.944 (0.154)	1.042 (0.187)	0.991 (0.128)	0.946 (0.169)	0.851 (0.213)	1.020 (0.237)	1.195 (0.188)
***R***^**2**^		.013***	.041***	.012***	.057***	.040***	.045***	.095***	.005***	.022***	.010***
**Marital status (*N*** **= 10,756)**											
Married/cohabiting	68.2	1.024 (0.111)	0.900 (0.179)	1.133 (0.156)	1.001 (0.153)	0.985 (0.205)	0.935 (0.151)	1.040 (0.188)	0.854 (0.219)	1.055 (0.242)	1.236 (0.196)
Divorced/widowed	14.0	1.031 (0.108)	0.916 (0.183)	1.113 (0.163)	0.990 (0.153)	0.995 (0.203)	0.941 (0.145)	1.038 (0.195)	0.838 (0.224)	1.087 (0.248)	1.220 (0.189)
Never married	17.8	1.021 (0.119)	0.882 (0.190)	1.110 (0.173)	1.034 (0.156)	0.930 (0.228)	0.907 (0.164)	1.108 (0.205)	0.873 (0.226)	1.112 (0.252)	1.241 (0.207)
***R***^**2**^		.001*	.003***	.004***	.007***	.011***	.005***	.018***	.002***	.008***	.001**
**Living situation (*N*** **= 10,739)**											
Alone	25.3	1.029 (0.117)	0.908 (0.186)	1.106 (0.174)	1.013 (0.160)	0.958 (0.222)	0.924 (0.160)	1.068 (0.214)	0.860 (0.229)	1.091 (0.256)	1.230 (0.206)
Two or more adults	41.4	1.029 (0.111)	0.919 (0.175)	1.126 (0.157)	1.000 (0.152)	0.992 (0.205)	0.947 (0.149)	1.012 (0.186)	0.845 (0.220)	1.042 (0.244)	1.230 (0.195)
Adult(s) and child(ren)	33.3	1.015 (0.109)	0.867 (0.183)	1.140 (0.154)	1.019 (0.150)	0.969 (0.204)	0.915 (0.149)	1.092 (0.178)	0.864 (0.216)	1.088 (0.236)	1.245 (0.191)
***R***^**2**^		.003***	.016***	.006***	.003***	.005***	.008***	.033***	.001***	.009***	.001**
**Education (*N*** **= 10,777)**											
Basic	5.9	1.012 (0.108)	0.913 (0.168)	1.122 (0.163)	0.998 (0.149)	0.976 (0.198)	0.942 (0.150)	1.003 (0.185)	0.925 (0.202)	1.130 (0.256)	1.192 (0.201)
Middle	37.8	1.010 (0.114)	0.886 (0.181)	1.139 (0.162)	1.024 (0.154)	0.967 (0.208)	0.917 (0.149)	1.053 (0.192)	0.890 (0.215)	1.110 (0.238)	1.237 (0.195)
Lower academic	32.6	1.030 (0.107)	0.905 (0.182)	1.123 (0.156)	1.007 (0.150)	0.985 (0.208)	0.932 (0.152)	1.048 (0.195)	0.835 (0.217)	1.062 (0.239)	1.242 (0.196)
Upper academic	23.7	1.042 (0.112)	0.908 (0.185)	1.109 (0.163)	1.010 (0.154)	0.979 (0.216)	0.948 (0.159)	1.070 (0.196)	0.811 (0.229)	1.001 (0.248)	1.233 (0.200)
***R***^**2**^		.013***	.003***	.005***	.006***	.001**	.006***	.006***	.027***	.032***	.003***
**HH income (*N*** **= 9989)**											
1st quartile	25.5	1.011 (0.118)	0.866 (0.187)	1.124 (0.162)	1.028 (0.154)	0.958 (0.215)	0.914 (0.153)	1.070 (0.199)	0.892 (0.218)	1.168 (0.241)	1.240 (0.204)
2nd quartile	32.9	1.024 (0.109)	0.898 (0.177)	1.120 (0.159)	1.012 (0.150)	0.974 (0.207)	0.930 (0.154)	1.052 (0.192)	0.863 (0.216)	1.082 (0.228)	1.233 (0.191)
3rd quartile	18.1	1.024 (0.108)	0.902 (0.176)	1.134 (0.158)	1.004 (0.155)	0.978 (0.205)	0.935 (0.148)	1.056 (0.190)	0.839 (0.216)	1.039 (0.227)	1.240 (0.199)
4th quartile	23.5	1.038 (0.112)	0.936 (0.180)	1.126 (0.165)	0.994 (0.156)	0.989 (0.214)	0.943 (0.156)	1.038 (0.198)	0.822 (0.221)	0.966 (0.247)	1.229 (0.198)
***R***^**2**^		.007***	.018***	.001*	.006***	.003***	.004***	.003***	.014***	.085***	<.001
**HH special diets (*N*** **= 10,503)**											
No special diet	52.8	1.019 (0.114)	0.907 (0.179)	1.135 (0.165)	1.009 (0.157)	0.962 (0.209)	0.918 (0.152)	1.059 (0.199)	0.867 (0.223)	1.087 (0.253)	1.242 (0.204)
Lactose-free	22.2	1.018 (0.103)	0.910 (0.171)	1.131 (0.149)	1.010 (0.149)	0.978 (0.202)	0.928 (0.146)	1.049 (0.187)	0.862 (0.210)	1.060 (0.234)	1.230 (0.192)
Gluten-free	7.0	1.050 (0.109)	0.863 (0.190)	1.111 (0.155)	1.015 (0.159)	1.024 (0.211)	0.939 (0.148)	1.025 (0.186)	0.852 (0.220)	1.040 (0.235)	1.224 (0.184)
Red-meat-free	5.1	1.052 (0.104)	0.898 (0.186)	1.092 (0.150)	0.994 (0.153)	1.029 (0.206)	0.977 (0.154)	1.012 (0.186)	0.792 (0.226)	1.018 (0.232)	1.209 (0.185)
Vegetarians	6.3	1.035 (0.119)	0.849 (0.196)	1.090 (0.168)	1.019 (0.144)	0.966 (0.225)	0.986 (0.154)	1.073 (0.188)	0.797 (0.216)	1.047 (0.244)	1.228 (0.184)
Other	6.5	1.024 (0.112)	0.870 (0.194)	1.112 (0.168)	1.014 (0.154)	1.001 (0.215)	0.941 (0.159)	1.056 (0.192)	0.847 (0.238)	1.058 (0.243)	1.238 (0.193)
***R***^**2**^		.009***	.011***	.008***	<.001	.010***	0.017***	.005***	.010***	.006***	.001**

*HE* health, *WC* weight control, *SA* sensory appeal, *MO* mood, *NC* natural content, *EC* ethical concern, *CO* convenience, *FA* familiarity, *PC* price-cheap, *PV* price-value, *HH* household

***P < .001, **P < .01, *P < .05

^a^Scores > 1 for each relative food motive indicate that the dimension is rated more important than on average all the motive items

^b^Number of participants with missing data for predictors in the analysed sample: age: 9, gender: 9, marital status: 39, living situation: 56, education: 18, household income: 121, household special diets: 292

^c^*R*^2^ values represent the proportion of the variance in the relative food motive that is explained by the given sociodemographic variable

## Results

**Fig. 1:**
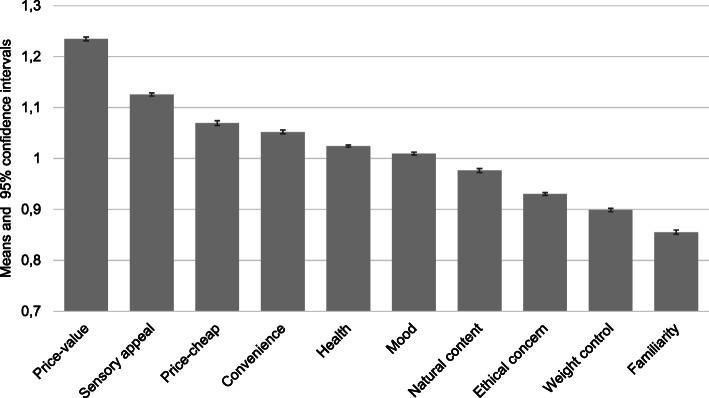
Mean values and 95% confidence intervals for relative food motives in the LoCard study (*N* = 10,795)

As can be seen from Table 1, 67% of the respondents were women, 45–64-year-olds formed the most common age group (38%; mean age being 46.3 years [SD = 14.8]), most of the respondents were married or cohabiting (68%), and the most typical living situation was “Two or more adults” (41%). The majority of the respondents had middle level (38%) or lower academic (33%) education. Monthly gross household incomes of 1500-2999€ and 3000-4499€ (21 and 23%, respectively) were the most typical income categories (before scaling for household size; not shown in Table 1). Half of the households had no special diet (53%), while 22% reported a lactose-free diet. Vegetarian and red-meat-free diets were followed by 6 and 5% of the households, respectively. In the entire sample, price-value was rated as the relatively most important motive guiding daily food selection, followed by sensory appeal, price-cheap, convenience and health motives (Fig. 1). Table 1 also shows the unadjusted mean values for each motive dimension by sociodemographic groups and associated *R*^2^ values. The strongest associations were observed between age and convenience (.095), household income (scaled) and price-cheap (.085), and age and mood control (.057). The younger the participants were, the more they valued convenience and mood control in their daily food selection. Participants with higher household income (scaled) considered cheapness less important than their lower-income counterparts.

Tables 2, 3 and 4 report estimates (including *R*^2^ change values) from the gender- and age-adjusted linear models separately for each motive dimension and sociodemographic predictor. In relation to appreciation of health motive, age group (.014), education (.013), and household diet (.011) had an increase in *R*^2^ value above .01 (Table 2). The regression coefficients indicated that the oldest age group (65–94) were more concerned about the health aspects of food than the youngest group (18–29). Likewise, participants with higher academic education valued health more than participants with basic, middle or lower academic education. Finally, households with gluten-free, vegetarian or red-meat-free diet valued health motive more than households without special diets. In relation to mood motive, only age group (.054) had *R*^2^ change value above the cut-off of .01. The older the participants were, the less they valued mood enhancing aspects of food. For convenience motive, living situation (.010) and age group (.093) stood out from the rest. Younger participants and households with adults and children valued convenience more than older participants and households with adults only.

**Table 2:** Results from multiple linear models predicting the relative importance of health, mood and convenience motive dimensions^a^

	Health				Mood				Convenience			
	B^b^	95% CI	P	∆*R*^2c^	B	95% CI	P	∆*R*^2^	B	95% CI	P	∆*R*^2^
**Gender**				.001***				.001**				<.001
Women	0.009	0.004, 0.013	<.001		0.010	0.004, 0.016	.001		−0.003	−0.010, 0.004	.43	
Men (ref.)												
**Age group**				.014***				.054***				.093***
18–29 (ref.)												
30–44	<0.001	−0.006, 0.007	.99		−0.043	−0.051, −0.034	<.001		−0.020	−0.031, −0.009	<.001	
45–64	0.009	0.002, 0.015	.007		−0.075	−0.084, −0.067	<.001		−0.103	−0.114, −0.093	<.001	
65–94	0.041	0.033, 0.049	<.001		−0.124	−0.135, −0.113	<.001		−0.177	−0.190, −0.164	<.001	
**Marital status**				<.001				<.001				.006***
Married/cohabiting (ref.)												
Divorced/widowed	<0.001	−0.006, 0.006	.99		0.001	−0.007, 0.010	.76		0.030	0.019, 0.040	<.001	
Never married	0.003	−0.002, 0.009	.26		0.005	−0.002, 0.013	.17		0.035	0.026, 0.045	<.001	
**Living situation**				.001**				.002***				.010***
Alone	0.008	0.002, 0.014	.006		0.015	0.008, 0.023	<.001		0.006	−0.003, 0.015	.21	
Two or more adults	0.007	0.002, 0.012	.010		0.012	0.005, 0.019	.001		−0.037	−0.046, −0.029	<.001	
Adult(s) and child(ren) (ref.)												
**Education**				.013***				.005***				.003***
Basic	−0.036	−0.046, −0.026	<.001		0.027	0.014, 0.040	<.001		−0.033	−0.049, −0.017	<.001	
Middle	−0.030	−0.036, −0.025	<.001		0.027	0.019, 0.034	<.001		−0.023	−0.032, −0.014	<.001	
Lower academic	−0.011	−0.017, −0.005	<.001		0.010	0.002, 0.017	.011		−0.028	−0.037, −0.018	<.001	
Upper academic (ref.)												
**Household income**				.005***				.001*				<.001
1st quartile	−0.027	−0.028, −0.015	<.001		0.009	<.001, 0.017	.046		−0.007	−0.018, 0.004	.19	
2nd quartile	−0.014	−0.020, −0.008	<.001		0.010	0.002, 0.018	.018		0.003	−0.007, 0.013	.60	
3rd quartile	−0.014	−0.018, −0.005	.001		0.001	−0.008, 0.010	.89		0.002	−0.009, 0.013	.74	
4th quartile (ref.)												
**Household special diets**				.011***				.001				.006***
No special diet (ref.)												
Lactose-free	<0.001	−0.005, 0.006	.95		−0.003	−0.010, 0.005	.46		−0.015	−0.024, −0.006	.001	
Gluten-free	0.032	0.024, 0.042	<.001		0.001	−0.011, 0.012	.89		−0.041	−0.055, −0.027	<.001	
Red-meat-free	0.033	0.023, 0.043	<.001		−0.017	−0.030, −0.003	.013		−0.048	−0.065, −0.032	<.001	
Vegetarians	0.023	0.014, 0.032	<.001		−0.013	−0.026, −0.001	.030		−0.022	−0.037, −0.007	.005	
Other	0.013	0.004, 0.022	.004		−0.007	−0.019, 0.005	.27		−0.020	−0.034, −0.005	.008	

***P < .001, **P < .01, *P < .05

^a^Each model includes age, gender and the given sociodemographic variable as predictors

^b^B values are unstandardized regression coefficients

^c^∆*R*^2^ refers to increase in the model *R*^2^ value after adding the given sociodemographic predictor to the age- and gender-adjusted model

**Table 3:** Results from multiple linear models predicting the relative importance of sensory appeal, weight control and natural content motive dimensions^a^

	Sensory appeal				Weight control				Natural content			
	B^b^	95% CI	P	∆*R*^2c^	B	95% CI	P	∆*R*^2^	B	95% CI	P	∆*R*^2^
**Gender**				.001**				<.001				.009***
Women	−0.011	−0.018, −0.005	.001		−0.001	−0.008, 0.007	.88		0.043	0.034, 0.051	<.001	
Men (ref.)												
**Age group**				.012***				.041***				.044***
18–29 (ref.)												
30–44	0.006	−0.004, 0.015	.25		0.006	−0.004, 0.017	.24		0.056	0.044, 0.068	<.001	
45–64	−0.003	−0.012, 0.006	.56		0.071	0.061, 0.081	<.001		0.101	0.089, 0.113	<.001	
65–94	−0.50	−0.062, −0.039	<.001		0.093	0.080, 0.106	<.001		0.149	0.134, 0.163	<.001	
**Marital status**				.005***				<.001				.004***
Married/cohabiting (ref.)												
Divorced/widowed	−0.009	−0.018, <0.001	.050		−0.004	−0.014, 0.006	.43		−0.018	−0.030, −0.007	.002	
Never married	−0.031	− 0.039, −0.023	<.001		0.004	−0.006, 0.013	.44		−0.034	−0.044, −0.023	<.001	
**Living situation**				.004***				.004***				.004***
Alone	−0.026	−0.034, −0.018	<.001		0.022	0.013, 0.031	<.001		−0.035	−0.046, −0.025	<.001	
Two or more adults	−0.003	− 0.011, 0.004	.37		0.026	0.018, 0.035	<.001		−0.010	−0.020, −0.001	.035	
Adult(s) and child(ren) (ref.)												
**Education**				.005***				.003***				.002***
Basic	0.021	0.007, 0.035	.003		−0.017	−0.033, −0.001	.032		−0.028	−0.046, −0.010	.002	
Middle	0.029	0.021, 0.037	<.001		−0.019	−0.028, −0.010	<.001		−0.009	−0.020, 0.001	.067	
Lower academic	0.013	0.005, 0.021	.001		0.001	−0.008, 0.010	.90		0.008	−0.002, 0.019	.12	
Upper academic (ref.)												
**Household income**				.001**				.008***				.001
1st quartile	−0.010	−0.020, −0.001	.027		−0.048	−0.058, −0.038	<.001		−0.008	−0.020, 0.004	.17	
2nd quartile	−0.008	−0.016, 0.001	.068		−0.032	−0.041, −0.022	<.001		−0.011	−0.022, <0.001	.045	
3rd quartile	0.004	−0.006, 0.014	.39		−0.025	−0.035, −0.014	<.001		−0.001	−0.014, 0.011	.86	
4th quartile (ref.)												
**Household special diets**				.010***				.007***				.011***
No special diet (ref.)												
Lactose-free	−0.006	−0.013, 0.002	.15		0.006	−0.002, 0.015	.17		0.018	0.008, 0.028	<.001	
Gluten-free	−0.026	−0.039, −0.014	<.001		−0.040	−0.053, −0.026	<.001		0.065	0.049, 0.081	<.001	
Red-meat-free	−0.044	−0.058, −0.030	<.001		−0.008	−0.024, 0.008	.32		0.066	0.048, 0.084	<.001	
Vegetarians	−0.054	−0.067, −0.041	<.001		−0.037	−0.051, −0.023	<.001		0.026	0.009, 0.043	.002	
Other	−0.027	−0.040, −0.015	<.001		−0.026	−0.040, −0.012	<.001		0.048	0.032, 0.065	<.001	

***P < .001, **P < .01, *P < .05

^a^Each model includes age, gender and the given sociodemographic variable as predictors

^b^B values are unstandardized regression coefficients

^c^∆*R*^2^ refers to increase in the model *R*^2^ value after adding the given sociodemographic predictor to the age- and gender-adjusted model

**Table 4:** Results from multiple linear models predicting the relative importance of ethical concern, familiarity and price motive dimensions^a^

	Ethical concern				Familiarity				Price-cheap				Price-value			
	B^b^	95% CI	P	∆*R*^2c^	B	95% CI	P	∆*R*^2^	B	95% CI	P	∆*R*^2^	B	95% CI	P	∆*R*^2^
**Gender**				.007***				.018***				.013***				.021***
Women	0.027	0.021, 0.033	<.001		−0.064	−0.073, − 0.055	<.001		− 0.061	−0.070, −0.051	<.001		−0.062	−0.069, −0.054	<.001	
Men (ref.)																
**Age group**				.049***				.007***				.026***				.015***
18–29 (ref.)																
30–44	0.034	0.025, 0.042	<.001		−0.022	−0.035, −0.010	.001		−0.062	−0.076, −0.048	<.001		− 0.022	−0.034, −0.011	<.001	
45–64	0.067	0.058, 0.075	<.001		−0.052	−0.065, −0.039	<.001		−0.101	−0.115, −0.087	<.001		−0.043	−0.054, −0.032	<.001	
65–94	0.116	0.106, 0.127	<.001		−0.046	−0.061, −0.030	<.001		−0.133	−0.150, −0.116	<.001		−0.083	−0.96, −0.069	<.001	
**Marital status**				.001***				<.001				.009***				<.001
Married/cohabiting (ref.)																
Divorced/widowed	−0.015	−0.024, −0.007	<.001		−0.002	−0.014, 0.011	.80		0.061	0.047, 0.074	<.001		0.004	−0.007, 0.015	.65	
Never married	−0.009	−0.017, −0.002	.019		0.013	0.001, 0.024	.027		0.038	0.026, 0.050	<.001		−0.006	−0.016, 0004	.26	
**Living situation**				.002***				.001*				.005***				<.001
Alone	−0.009	−0.017, −0.002	.019		0.002	−0.009, 0.014	.67		0.022	0.010, 0.034	<.001		−0.003	−0.013, 0.007	.52	
Two or more adults	0.007	<0.001, 0.014	.051		−0.010	−0.21, <0.001	.044		−0.021	−0.032, −0.010	<.001		0.001	−0.008, 0.010	.86	
Adult(s) and child(ren) (ref.)																
**Education**				.005***				.029***				.035***				.002***
Basic	−0.024	−0.037, −0.012	<.001		0.122	0.103, 0.141	<.001		0.150	0.129, 0.170	<.001		−0.030	−0.047, −0.013	<.001	
Middle	−0.028	−0.036, −0.021	<.001		0.080	0.069, 0.090	<.001		0.107	0.095, 0.119	<.001		0.004	−0.006, 0.013	.46	
Lower academic	−0.014	−0.021, −0.006	<.001		0.026	0.015, 0.037	<.001		0.059	0.047, 0.071	<.001		0.009	−0.001, 0.019	.079	
Upper academic (ref.)																
**Household income**				.001*				.014***				.077***				<.001
1st quartile	−0.009	−0.018, −0.001	.032		0.072	0.060, 0.085	<.001		0.195	0.182, 0.208	<.001		0.004	−0.007, 0.015	.49	
2nd quartile	−0.009	−0.017, −0.002	.019		0.047	0.035, 0.058	<.001		0.120	0.107, 0.132	<.001		0.007	−0.004, 0.017	.21	
3rd quartile	0.001	−0.009, 0.010	.91		0.017	0.004, 0.030	.013		0.069	0.055, 0.083	<.001		0.008	−0.004, 0.020	.21	
4th quartile (ref.)																
**Household special diets**				.024***				.010***				.008***				.002**
No special diet (ref.)																
Lactose-free	0.012	0.005, 0.019	.001		−0.003	−0.014, 0.007	.52		−0.027	−0.039, −0.016	<.001		−0.011	−0.020, −0.002	.021	
Gluten-free	0.024	0.013, 0.035	<.001		−0.014	−0.030, 0.003	.11		−0.048	−0.067, −0.030	<.001		−0.017	−0.032, −0.002	.025	
Red-meat-free	0.059	0.046, 0.072	<.001		−0.072	−0.091, −0.053	<.001		− 0.067	−0.088, −0.045	<.001		−0.029	−0.047, −0.012	.001	
Vegetarians	0.087	0.075, 0.099	<.001		−0.074	−0.091, −0.056	<.001		− 0.057	−0.076, −0.037	<.001		− 0.021	−0.037, −0.005	.009	
Other	0.031	0.019, 0.043	<.001		−0.019	−0.036, −0.001	.036		−0.035	−0.054, −0.016	<.001		−0.004	−0.020, 0.011	.60	

***P < .001, **P < .01, *P < .05

^a^Each model includes age, gender and the given sociodemographic variable as predictors

^b^B values are unstandardized regression coefficients

^c^∆*R*^2^ refers to increase in the model *R*^2^ value after adding the given sociodemographic predictor to the age- and gender-adjusted model

Best predictors for sensory appeal motive were age group (.012) and special diets in household (.010) (Table 3). People in the oldest age group seemed to care less about the sensory pleasures related to food than participants belonging to the youngest age group. Also, households without any special diets were more concerned about sensory appeal motive than households with gluten-free, red-meat-free, vegetarian, or other type of diet. In relation to weight control motive, age group (.041) was the only sociodemographic predictor with *R*^2^ change exceeding .01. Respondents aged 45 or more valued weight control motive more than respondents belonging to the youngest age group (18–29). In regard to natural content motive, age group (.044) and special diets in household (.011) were the best predictors. A descending trend was observed for age: the younger the participants were the less they cared about natural content motive. Likewise, households without any special diets placed less value on natural content motive than households where lactose-free, gluten-free, red-meat-free, or other type of special diet was followed.

In relation to ethical concern motive, again, age group (.049) and special diets in household (.024) proved out to be the best sociodemographic predictors (Table 4). The older the participants were, the more they valued ethical concern motive. Also, households following gluten-free, red-meat-free, vegetarian, or other type of special diet placed more value on ethical concern motive than households without any special diets. In regard to familiarity motive, gender (.018), education (.029), household income (.014), and special diets in household (.010) all had *R*^2^ change value above .01. Women appreciated less familiarity of food than men. Participants with higher academic education also cared less about familiarity than those with lower academic, middle, or basic education. Likewise, respondents in the highest income quartile were less concerned about familiarity than people in the two lowest income quartiles. Finally, households following a vegetarian or red-meat-free diet valued less familiarity than households where no special diet was followed.

Best predictors for price-cheap motive were gender (.013), age group (.026), education (.035), and household income (.077) (Table 4). Men appreciated cheapness of food more than women. Also, the younger the participants were, the more they valued price-cheap motive. Similar ascending trend was observed in regard to education: the less educated the participants were, the more they valued inexpensiveness of food. Not surprisingly, also household income showed a similar pattern: participants in the lower quartiles valued inexpensiveness more than those in the highest quartile. In regard to price-value motive, gender (.021) and age group (.015) had the highest increase in *R*^2^ change estimate. Men appreciated more price-value motive than women. Also, an ascending trend was observed for age: respondents in the youngest age group appreciated price-value motive more than those in the older age groups.

**Fig. 2:**
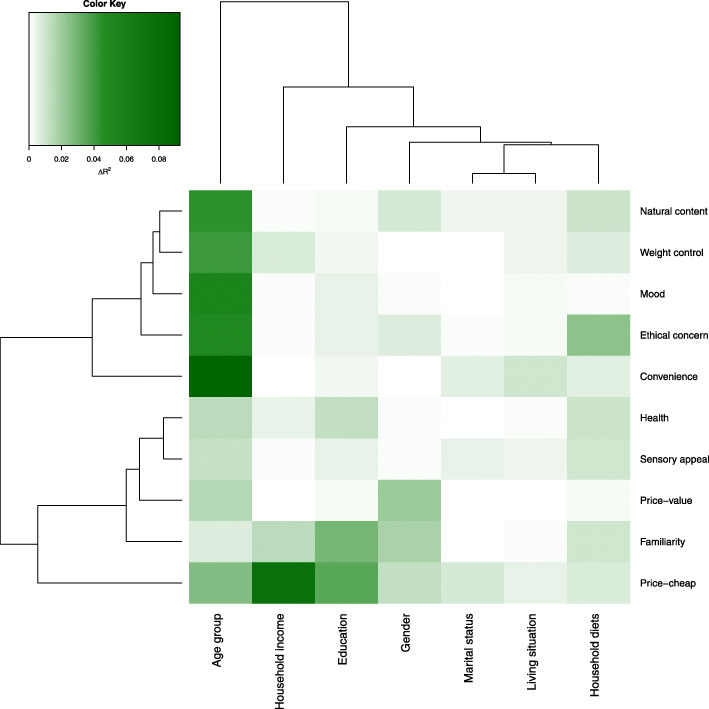
Heatmap visualizing the variation in the strength of the associations between sociodemographic factors and relative food motives. The ∆*R*^2^ values represent increase in the proportion of variance explained in each motive dimension after adding the sociodemographic predictor to the age- and gender-adjusted linear model. Motives and sociodemographic predictors (rows and columns) are arranged according to the similarity in ∆*R*^2^

Finally, the heatmap (Fig. 2) illustrates that age stood out as having the strongest associations with convenience, ethical concern, mood, weight control and natural content motives. Overall, the changes in *R*^2^ due to inclusion of marital status or living situation were smaller than for other predictors. Household special diets showed a somewhat distinct pattern where the association with ethical concern was most pronounced.

## Discussion

The present research provided detailed and up-to-date knowledge on how the relative importance of food motives varies across different segments of the population. Age and household income occurred as the sociodemographic factors that showed the strongest associations with certain food motives: young adults placed greater emphasis on convenience and mood control than middle-aged and older adults, while the salience of cheapness decreased with increasing household income. Albeit somewhat weaker in strength, we also detected several other links between sociodemographic characteristics, special diets and individuals’ motive priorities as discussed in the following paragraphs.

In accordance with a few previous studies [9, 11], age emerged as a central determinant of motives for food selection. Overall, our findings are consistent with the suggestion [11] that older individuals may value more “long-term-oriented” motives (health, weight control, natural content, ethical concern), while younger people may emphasize more “short-term-oriented” motives (mood, sensory appeal, price, convenience). The emergence of diverse health problems and the phase of the life course, such as having children and more stabilized life and financial situation, are likely to contribute to more long-term and health-conscious orientation in middle-aged and older adults. The observation that considerations related to mood control (e.g. helps me cope with stress) were most salient to 18–29-year-olds could also reflect the increased rates of frequent psychological distress in Finnish young adults [29] and the impact of distress in those prone to emotional eating [30]. Even though younger people often report being more environmentally concerned than older people [31], the present and previous studies [9, 15] applying the FCQ have found a positive association between age and ethical concern motive. However, one potential explanation for this discrepancy is that the ethical concern dimension contained only one item (“is packaged in an environmentally friendly way”) assessing wider environmental aspects.

Gender differences in the examined food motives were substantially more pronounced in the absolute than in the relative terms; a phenomenon that was also observed in a population-based study of Finnish working-age adults [12]. Consistent with earlier studies [9, 11, 12, 14, 15] analysing gender differences in the absolute terms, we found that women rated the absolute importance of health, ethical concern, natural content, mood, sensory appeal, convenience and weight control as higher than men. This probably reflects that women still have the main responsibility for grocery shopping and cooking in many households [32] and are exposed to stricter sociocultural norms for body shape [33] with both phenomena leading to greater involvement and preoccupation with food in females. In contrast, gender differences in the relative importance of motives were mostly weak in line with the fact that relative motives do not capture individual differences in the level of involvement with food [12]. Nonetheless, a novel and potentially important observation was that men rated the relative importance of price-value, familiarity and price-cheap as higher than women; a pattern reflecting a more practical motivational orientation in males.

Price, familiarity and health emerged as the motive dimensions that most notably differentiated between education and household income categories: participants with higher SEP valued more healthiness and less cheapness and familiarity in their daily food selection compared to lower-SEP participants. A similar pattern has also been reported in earlier studies [12, 16–20], but a unique finding in our study was that the price item (“is good value for money”) depicting the concept of worth did not distinguish between SEP categories. Food being good value for money actually emerged as the most important motive (in both absolute and relative terms) in all education and household income groups, which suggests that the impact of the price-value considerations on food selection is dominant and widespread. It is yet possible that there are systematic SEP differences in what kind of foods and portion sizes fulfill the criteria of being good value for money; a topic to be addressed in future studies. We further observed that the positive relationship between education and health motive was more prominent than the one involving household income. Albeit education and income are closely intertwined, they may influence the importance of health considerations via separate pathways. On the one hand, spending longer time in the educational system may improve nutrition and food literacy as well as socialize individuals to adopt healthy dietary patterns [34]. On the other hand, affluent individuals and households possess greater financial freedom to take the health aspects into account given that foods of higher nutritional quality have been demonstrated to cost more per calorie [35]. Besides these separate pathways, the stronger link between education and health motive can also reflect the fact that both of them were measured at the individual level, while income was assessed at the household level.

Higher education and household income were both linked to a lower salience of familiarity in daily food selection (albeit education again with a stronger effect size), and these associations are probably driven by diverse mechanisms. Lower economic resources may act as a barrier for eating unfamiliar foods: experimenting with new foods and dishes often involves a risk of waste that less affluent individuals cannot afford to take [36]. The mechanisms of social distinction [37] may also play a role: individuals with higher SEP can be more willing to incorporate new foods in their diets because it provides one medium to set themselves apart from the other SEP groups [38, 39]. It has indeed been proposed that health-related lifestyle has become increasingly important source of social distinction in the present prosperous societies where more people can afford various consumer goods [39].

There is rather little previous evidence on whether motives for food selection differ by marital status, living situation and/or having children. An Irish study found that singles rated natural content, weight control and sensory appeal slightly lower than those with partner, while there was a small positive association between having children and price [15]. In our study, the impact of marital status and living situation on individuals’ motive priorities tended to be weak with some effects observed on convenience and price-cheap motives: participants living with child(ren) tended to emphasize convenience and unmarried participants appreciated cheapness. However, to fully understand how diverse living situations influence food motives, future research with more detailed groupings (e.g. based on the respondent’s gender and the number and age of children living in the household) is needed.

Although a lactose-free diet was the most common special diet in the studied households (22% of the households), our main interest concerned households following more plant-based diets. All in all, respondents living in households with vegetarian and red-meat-free diets appeared to possess a more ethically- and health-conscious motivational orientation compared to households with no special diets. A few studies assessing diets on an individual level have reported parallel observations [10, 40]. Furthermore, familiarity and sensory appeal emerged as additional motives that distinguished between households with vegetarian/red-meat-free diets and no special diets implying that these motives have a potential to act as barriers to following a more plant-based diet. Though we were unable to distinguish whether the reported diets were actually followed by the respondent or other household members, a special diet in the household is prone to influence all members’ diets as eating patterns have been found to be socially transmissible across various kinds of relationships, including spouses and parent-child dyads [10, 41]. It is noteworthy that the causal links between special diets and food motives are potentially more complex than the ones involving sociodemographic factors. It is plausible to assume that age, gender, SEP, marital status and living situation as well as related experiences and resources influence individual’s motive priorities rather than vice versa. In contrast, prioritizing certain motives can lead to adopting a specific diet, while following the diet can also gradually affect the relative importance of motives.

### Practical implications

The present study offers two complementary areas of practical implications. First, our findings contribute to the knowledge-base that helps especially scholars, nutrition professionals and policy-makers to design more efficient and tailored interventions promoting diets that benefit human and planetary health across sociodemographic categories. To ensure the maintenance of dietary changes, it is vital to develop and implement actions that allow individuals to incorporate new eating practices in such a way that simultaneously satisfy a range of motives important in their food selection. For instance, food-related interventions targeting increased vegetable and reduced red meat intakes in lower-SEP individuals and men can benefit from taking into account greater priority of familiarity and price among other issues. A concrete example of this type of intervention could entail close collaboration with lunch or workplace canteens to develop familiar food recipes by replacing a part of red meat with plant-based protein while simultaneously ensuring the affordability and tastiness. Given that one of the strongest associations occurred between lower household income and higher salience of cheapness, our results also support the potential of using price interventions to reduce socioeconomic inequalities in healthy eating [8].

Second, Vadakkepatt et al. [42] have recently put forth the need for “sustainable retailing” to recognize and emphasize food retailers’ role in addressing the manyfold challenges related to sustainability and health. Given their unique position in the food supply chain, retailers have a critical role and potential in encouraging and facilitating changes in consumer behaviour towards healthier and more sustainable future. Toward that end, detailed insights on how food motives, such as price and convenience, relate to specific sociodemographic factors can enrich food retailers’ understanding of consumer preferences, and help them support consumers’ behavioural changes through more effective segmentation and promotional activities. This knowledge can likewise help food manufacturers to facilitate behavioural changes by designing products that, for example, take the role of familiarity for specific consumer segments better into account.

### Strengths and limitations

The main strength of this study is that we utilized recently collected data of more than 10,000 Finnish adults with information on a diverse set of sociodemographic characteristics and food motives. Moreover, a valuable and unique contribution is that we simultaneously focused on the relative importance of motives (instead of only analysing the absolute importance of each motive) because it may better reflect the complexity of the motive structure. In line with this interpretation, the associations between food motives and dietary intake (as measured by a food frequency questionnaire) were found to be weaker on the absolute than on the relative level [12]. Nonetheless, we included estimates for absolute motives in additional material (see Additional file 2) to retain the full comparability between our research and previous ones using the FCQ [9].

A limitation is that compared to the general Finnish adult population, the sample included more women, employed individuals and those with higher education, while retired individuals and those aged under 30 and over 70 years were underrepresented [26]. However, sensitivity analyses applying post-stratification weights developed to correct this bias [26] produced similar results with a trend that the associations between age and food motives became somewhat stronger (see Additional file 3). Even though the cross-sectional study design does not allow conclusions to be drawn on causality, it can be argued that sociodemographic characteristics are more likely to influence food motives than vice versa. Because the high number of statistical tests conducted increased the likelihood for Type 1 errors, we decided to emphasize the strength of the associations (as indicated by ∆*R*^2^ values) when reporting and interpreting the results. Accordingly, we gave more emphasis on stronger associations with significance markedly smaller than the conventional nominal level of statistical significance (5%).

There are also potential limitations in the utilized measures due to the restricted questionnaire length. Special diets and income were measured only at the household level as discussed above. Although we assessed numerous motive dimensions using the 28-item version of the FCQ developed in 1995 [9], our study is somewhat restricted in reflecting the most recent developments in norms regarding motives for food selection (e.g. motives reflecting changed norms for locally produced foods or varied forms of masculinity [43]). Moreover, it would have been beneficial to measure price, familiarity and natural content more comprehensively with a higher number of items.

## Conclusions

Our findings imply that sociodemographic differences in a range of motives for food selection (including familiarity, price, mood control and convenience) exist that might act as barriers or drivers for adopting a diet that is characterized by increased vegetable intake as well as by reduced red meat intake. Interventions aiming to diminish SEP and gender disparities in the healthiness and sustainability of diets should employ strategies that take into account higher priority of familiarity and price in daily food selection in lower-SEP individuals and males.

## Supplementary Information

**Additional file 1.** Results from exploratory factor analysis of the 28-item Food Choice Questionnaire.

**Additional file 2: Table 1.** Mean values for absolute food motives by sociodemographic groups. **Table 2.** Results from multiple linear models predicting the absolute importance of health, mood and convenience motive dimensions. **Table 3.** Results from multiple linear models predicting the absolute importance of sensory appeal, weight control and natural content motive dimensions. **Table 4.** Results from multiple linear models predicting the absolute importance of ethical concern, familiarity and price motive dimensions.

**Additional file 3:** **Table 1.** Results from re-weighted multiple linear models predicting the relative importance of health, mood and convenience motive dimensions. **Table 2.** Results from re-weighted multiple linear models predicting the relative importance of sensory appeal, weight control and natural content motive dimensions. **Table 3.** Results from re-weighted multiple linear models predicting the relative importance of ethical concern, familiarity and price motive dimensions.

## Data Availability

Data are owned by a third party (S Group) and were used under a research agreement for the current study, and are not publicly available. According to the research agreement, the authors are not allowed to share the data.

## Abbreviations

FCQ: Food Choice Questionnaire
LoCard study: Loyalty Card study
SEP: Socioeconomic position

## References

[1] Petrovic D, de Mestral C, Bochud M, Bartley M, Kivimaki M, Vineis P (2018). The contribution of health behaviors to socioeconomic inequalities in health: a systematic review. Prev Med.

[2] Darmon N, Drewnowski A (2008). Does social class predict diet quality?. Am J Clin Nutr.

[3] Giskes K, Avendano M, Brug J, Kunst AE (2010). A systematic review of studies on socioeconomic inequalities in dietary intakes associated with weight gain and overweight/obesity conducted among European adults. Obes Rev.

[4] Clonan A, Roberts KE, Holdsworth M (2016). Socioeconomic and demographic drivers of red and processed meat consumption: implications for health and environmental sustainability. Proc Nutr Soc.

[5] Valsta L, Kaartinen N, Tapanainen H, Männistö S, Sääksjärvi K (2018). Ravitsemus Suomessa - FinRavinto 2017 -tutkimus [nutrition in Finland - the national FinDiet 2017 survey].

[6] Hosseinpoor AR, Bergen N, Schlotheuber A (2015). Promoting health equity: WHO health inequality monitoring at global and national levels. Glob Health Action.

[7] Willett W, Rockstrom J, Loken B, Springmann M, Lang T, Vermeulen S (2019). Food in the Anthropocene: the EAT-lancet commission on healthy diets from sustainable food systems. Lancet..

[8] McGill R, Anwar E, Orton L, Bromley H, Lloyd-Williams F, O'Flaherty M (2015). Are interventions to promote healthy eating equally effective for all? Systematic review of socioeconomic inequalities in impact. BMC Public Health.

[9] Steptoe A, Pollard TM, Wardle J (1995). Development of a measure of the motives underlying the selection of food: the food choice questionnaire. Appetite..

[10] Pollard TM, Steptoe A, Wardle J (1998). Motives underlying healthy eating: using the food choice questionnaire to explain variation in dietary intake. J Biosoc Sci.

[11] Renner B, Sproesser G, Strohbach S, Schupp HT (2012). Why we eat what we eat. The Eating Motivation Survey (TEMS). Appetite.

[12] Konttinen H, Sarlio-Lahteenkorva S, Silventoinen K, Mannisto S, Haukkala A (2013). Socio-economic disparities in the consumption of vegetables, fruit and energy-dense foods: the role of motive priorities. Public Health Nutr.

[13] Alles B, Peneau S, Kesse-Guyot E, Baudry J, Hercberg S, Mejean C (2017). Food choice motives including sustainability during purchasing are associated with a healthy dietary pattern in French adults. Nutr J.

[14] Glanz K, Basil M, Maibach E, Goldberg J, Snyder D (1998). Why Americans eat what they do: taste, nutrition, cost, convenience, and weight control concerns as influences on food consumption. J Am Diet Assoc.

[15] Schliemann D, Woodside JV, Geaney F, Cardwell C, McKinley MC, Perry I (2019). Do socio-demographic and anthropometric characteristics predict food choice motives in an Irish working population?. Br J Nutr.

[16] Lennernas M, Fjellstrom C, Becker W, Giachetti I, Schmitt A, Remaut de Winter A (1997). Influences on food choice perceived to be important by nationally-representative samples of adults in the European Union. Eur J Clin Nutr.

[17] Steptoe A, Wardle J (1999). Motivational factors as mediators of socioeconomic variations in dietary intake patterns. Psychol Health.

[18] Hupkens CLH, Knibbe RA, Drop MJ (2000). Social class differences in food consumption. The explanatory value of permissiveness and health and cost considerations. Eur J Pub Health.

[19] Bowman SA (2006). A comparison of the socioeconomic characteristics, dietary practices, and health status of women food shoppers with different food price attitudes. Nutr Res.

[20] Pechey R, Monsivais P, Ng Y, Marteau TM (2015). Why don't poor men eat fruit? Socioeconomic differences in motivations for fruit consumption. Appetite..

[21] Kaminski M, Skonieczna-Zydecka K, Nowak JK, Stachowska E (2020). Global and local diet popularity rankings, their secular trends, and seasonal variation in Google Trends data. Nutrition.

[22] Sobal J, Bisogni CA, Devine CM, Jastran M, Shepherd R, Raats M (2006). A conceptual model of the food choice process over the life course. The psychology of food choice. Frontiers in nutritional science, no.

[23] Sobal J, Bisogni CA (2009). Constructing food choice decisions. Ann Behav Med.

[24] Lappalainen R, Saba A, Holm L, Mykkanen H, Gibney MJ, Moles A (1997). Difficulties in trying to eat healthier: descriptive analysis of perceived barriers for healthy eating. Eur J Clin Nutr.

[25] Scheibehenne B, Miesler L, Todd PM (2007). Fast and frugal food choices: uncovering individual decision heuristics. Appetite..

[26] Vuorinen AL, Erkkola M, Fogelhom M, Kinnunen S, Saarijarvi H, Uusitalo L, et al. Characterization and correction of bias due to nonparticipation and the degree of loyalty in large-scale Finnish loyalty card data on grocery purchases. J Med Internet Res. 2020. 10.2196/18059. 10.2196/18059 PMC7392131 32459633

[27] Cohen J (1988). Statistical power analysis for the behavioral sciences.

[28] Organisation for Economic Co-operation and Development (OECD). The OECD List of Social Indicators: Paris, OECD Social Indicator Development Programme; 1982.

[29] Oksanen A, Laimi K, Bjorklund K, Loyttyniemi E, Kunttu K (2017). a 12-year trend of psychological distress: national study of Finnish university students. Cent Eur J Public Health.

[30] Konttinen H. Emotional eating and obesity in adults: the role of depression, sleep and genes. Proc Nutr Soc. 2020:1–7. 10.1017/S0029665120000166. 10.1017/S0029665120000166 32213213

[31] Gifford R, Nilsson A (2014). Personal and social factors that influence pro-environmental concern and behaviour: a review. Int J Psychol.

[32] Holm L, Ekström MP, Hach S, Lund TB (2015). Who is cooking dinner? Changes in the gendering of cooking from 1997 to 2012 in four Nordic countries. Food, Cult Soc.

[33] Buote VM, Wilson AE, Strahan EJ, Gazzola SB, Papps F (2011). Setting the bar: divergent sociocultural norms for women's and men's ideal appearance in real-world contexts. Body Image.

[34] Yen IH, Moss N (1999). Unbundling education: a critical discussion of what education confers and how it lowers risk for disease and death. Ann N Y Acad Sci.

[35] Darmon N, Drewnowski A (2015). Contribution of food prices and diet cost to socioeconomic disparities in diet quality and health: a systematic review and analysis. Nutr Rev.

[36] Barker M, Lawrence WT, Skinner TC, Haslam CO, Robinson SM, Inskip HM, Margetts BM, Jackson AA, Barker DJP, Cooper C, the Food Choice Group, University of Southampton (2008). Constraints on food choices of women in the UK with lower educational attainment. Public Health Nutr.

[37] Bourdieu P (1984). Distinction: a social critique of the judgement of taste.

[38] Pampel FC, Krueger PM, Denney JT (2010). Socioeconomic disparities in health behaviors. Annu Rev Sociol.

[39] Oude Groeniger J, van Lenthe FJ, Beenackers MA, Kamphuis CB (2017). Does social distinction contribute to socioeconomic inequalities in diet: the case of ‘superfoods’ consumption. Int J Behav Nutr Phys Act.

[40] Vainio A, Niva M, Jallinoja P, Latvala T (2016). From beef to beans: Eating motives and the replacement of animal proteins with plant proteins among Finnish consumers. Appetite.

[41] Vepsalainen H, Nevalainen J, Fogelholm M, Korkalo L, Roos E, Ray C (2018). Like parent, like child? Dietary resemblance in families. Int J Behav Nutr Phys Act.

[42] Vadakkepatt GG, Page Winterich K, Mittal V, Zinn W, Beitelspacher L, Aloysius J (2021). Sustainable retailing. J Retail.

[43] De Backer C, Erreygers S, De Cort C, Vandermoere F, Dhoest A, Vrinten J (2020). Meat and masculinities. Can differences in masculinity predict meat consumption, intentions to reduce meat and attitudes towards vegetarians?. Appetite.

