# Supplementary material for: Sociodemographic differences in motives for food selection: results from the LoCard cross-sectional survey
Source: Int J Behav Nutr Phys Act. 2021 Jun 2;18:71. doi: 10.1186/s12966-021-01139-2 (PMC8173871; doi:10.1186/s12966-021-01139-2)
Supplement: Supplementary file 1 — Additional file 1. Results from exploratory factor analysis of the 28-item Food Choice Questionnaire. [file 12966_2021_1139_MOESM1_ESM.docx]

Additional file 1. Results from exploratory factor analysis of the 28-item Food Choice Questionnaire

| **Variable** | **Factor1** | **Factor2** | **Factor3** | **Factor4** | **Factor5** | **Factor6** | **Factor7** | **Factor8** | **Factor9** |
| --- | --- | --- | --- | --- | --- | --- | --- | --- | --- |
| Fairtrade | **.792** | .013 | -.009 | -.011 | -.021 | .060 | -.004 | -.027 | .079 |
| Organically grown | **.680** | -.026 | -.041 | -.022 | .016 | -.164 | .016 | .071 | .083 |
| Env. friendly package | **.642** | -.003 | .029 | .038 | -.052 | -.072 | -.050 | .071 | -.042 |
| Countries politically approved | **.612** | -.027 | .027 | .053 | -.028 | -.005 | -.042 | -.024 | -.082 |
| Domestically produced | **.341** | .014 | .048 | .060 | .133 | -.232 | .015 | -.015 | -.087 |
| Easy prepare | -.013 | **.898** | -.019 | -.008 | -.066 | -.039 | .016 | -.031 | -.020 |
| No time prepare | -.012 | **.825** | .006 | .001 | .002 | .036 | -.022 | -.015 | -.015 |
| Smells nice | -.004 | -.009 | **.842** | .005 | .016 | -.026 | .015 | -.072 | -.018 |
| Makes me feel good | .036 | .011 | **.656** | -.052 | -.004 | .055 | -.103 | -.037 | .136 |
| Looks nice | .040 | -.009 | **.557** | .032 | .114 | -.001 | -.031 | .050 | -.056 |
| Tastes good | -.046 | -.008 | **.474** | .000 | -.091 | -.048 | .032 | .148 | -.036 |
| Low fat | .031 | -.013 | .031 | **.801** | .043 | .001 | .011 | -.002 | -.141 |
| Low calories | .037 | .024 | -.037 | **.787** | -.038 | -.058 | .042 | .021 | .106 |
| Helps weight control | -.028 | -.025 | -.033 | **.478** | -.016 | .017 | -.339 | .042 | .287 |
| Usually eat | -.024 | -.030 | -.022 | -.030 | **.782** | -.010 | -.030 | -.018 | .026 |
| Is familiar | -.039 | -.014 | .027 | .029 | **.774** | -.006 | .042 | .002 | -.025 |
| Shops close I live | .102 | .193 | -.009 | -.053 | **.263** | .053 | -.055 | .228 | .000 |
| No additives | -.013 | .031 | -.007 | .005 | -.017 | **-.887** | -.036 | -.030 | .053 |
| No artificial ingredients | .148 | -.075 | .036 | .020 | .030 | **-.602** | -.124 | .016 | -.039 |
| Keeps me healthy | -.005 | .048 | -.052 | -.020 | -.050 | -.080 | **-.679** | .137 | -.003 |
| Contains vitamins | .154 | -.014 | .006 | -.021 | -.014 | -.081 | **-.636** | -.018 | -.159 |
| Keeps me awake | -.029 | .007 | .112 | -.010 | -.017 | -.051 | **-.620** | .042 | .175 |
| Is good for skin | .078 | -.012 | .059 | -.022 | .055 | -.077 | **-.603** | .002 | .025 |
| High in protein | .013 | .025 | .046 | .142 | .077 | .005 | **-.415** | -.007 | .005 |
| High in fibre | .106 | .017 | .069 | .341 | .000 | -.028 | **-.392** | -.091 | -.222 |
| Good value | .068 | -.051 | .090 | -.025 | -.039 | .009 | -.094 | **.620** | -.044 |
| Is cheap | -.066 | .111 | .023 | .104 | .114 | -.008 | .014 | **.265** | .067 |
| Cope with stress | .109 | .104 | .318 | .015 | .094 | .013 | -.057 | -.049 | **.326** |

Forced nine-factor solution, extracted with maximum likelihood and rotated with oblimin rotation (with Kaiser normalization). Total variance explained 51.49%.
