# Supplementary material for: Sociodemographic differences in motives for food selection: results from the LoCard cross-sectional survey
Source: Int J Behav Nutr Phys Act. 2021 Jun 2;18:71. doi: 10.1186/s12966-021-01139-2 (PMC8173871; doi:10.1186/s12966-021-01139-2)
Supplement: Supplementary file 2 — Additional file 2: Table 1. Mean values for absolute food motives by sociodemographic groups. Table 2. Results from multiple linear models predicting the absolute importance of health, mood and convenience motive dimensions. Table 3. Results from multiple linear models predicting the absolute importance of sensory appeal, weight control and natural content motive dimensions. Table 4. Results from multiple linear models predicting the absolute importance of ethical concern, familiarity and price motive dimensions. [file 12966_2021_1139_MOESM2_ESM.docx]

Additional file 2. Table 1. Mean values for absolute food motives by sociodemographic groups

|  | HE | WC | SA | MO | NC | EC | CO | FA | PC | PV |
| --- | --- | --- | --- | --- | --- | --- | --- | --- | --- | --- |
|  | Mean (SD) | Mean (SD) | Mean (SD) | Mean (SD) | Mean (SD) | Mean (SD) | Mean (SD) | Mean (SD) | Mean (SD) | Mean (SD) |
| **Gender, R^2a^** | .033*** | .009*** | .033*** | .042*** | .025*** | .027*** | .045*** | <.001 | .003*** | .008*** |
| Women | 3.076 (0.493) | 2.690 (0.630) | 3.343 (0.493) | 3.036 (0.544) | 2.964 (0.700) | 2.808 (0.568) | 3.136 (0.531) | 2.492 (0.672) | 3.128 (0.675) | 3.615 (0.510) |
| Men | 2.878 (0.532) | 2.560 (0.642) | 3.148 (0.519) | 2.789 (0.581) | 2.718 (0.767) | 2.602 (0.617) | 2.891 (0.538) | 2.500 (0.650) | 3.052 (0.692) | 3.515 (0.554) |
|  |  |  |  |  |  |  |  |  |  |  |
| **Age group, R^2^** | .016*** | .045*** | .003*** | .020*** | .040*** | .042*** | .067*** | .003*** | .015*** | .003*** |
| 18-29 | 2.962 (0.526) | 2.501 (0.651) | 3.273 (0.524) | 3.105 (0.519) | 2.646 (0.762) | 2.562 (0.591) | 3.236 (0.505) | 2.554 (0.656) | 3.291 (0.617) | 3.641 (0.504) |
| 30-44 | 2.951 (0.513) | 2.508 (0.627) | 3.277 (0.506) | 2.975 (0.550) | 2.785 (0.736) | 2.655 (0.599) | 3.169 (0.515) | 2.497 (0.658) | 3.111 (0.678) | 3.578 (0.534) |
| 45-64 | 3.023 (0.508) | 2.741 (0.607) | 3.305 (0.498) | 2.929 (0.579) | 2.949 (0.699) | 2.787 (0.576) | 2.987 (0.544) | 2.457 (0.665) | 3.059 (0.690) | 3.582 (0.522) |
| 65-94 | 3.147 (0.501) | 2.832 (0.584) | 3.212 (0.528) | 2.817 (0.588) | 3.122 (0.683) | 2.958 (0.538) | 2.809 (0.530) | 2.531 (0.679) | 3.017 (0.694) | 3.534 (0.542) |
|  |  |  |  |  |  |  |  |  |  |  |
| **Marital status, R^2^** | .002*** | .004*** | .003*** | .002*** | .009*** | .005*** | .014*** | .001* | .008*** | <.001 |
| Married/cohabiting | 3.002 (0.508) | 2.641 (0.616) | 3.292 (0.497) | 2.942 (0.565) | 2.899 (0.715) | 2.744 (0.584) | 3.019 (0.539) | 2.489 (0.657) | 3.059 (0.680) | 3.581 (0.528) |
| Divorced/widowed | 3.070 (0.528) | 2.735 (0.643) | 3.285 (0.537) | 2.938 (0.590) | 2.972 (0.723) | 2.809 (0.587) | 3.045 (0.562) | 2.477 (0.686) | 3.190 (0.688) | 3.588 (0.518) |
| Never married | 2.991 (0.532) | 2.590 (0.666) | 3.217 (0.533) | 3.006 (0.563) | 2.740 (0.787) | 2.662 (0.621) | 3.191 (0.541) | 2.531 (0.677) | 3.199 (0.671) | 3.578 (0.533) |
|  |  |  |  |  |  |  |  |  |  |  |
| **Living situation, R^2^** | .004*** | .015*** | .003*** | .001** | .005*** | .008*** | .027*** | .001* | .006*** | <.001 |
| Alone | 3.033 (0.542) | 2.684 (0.660) | 3.230 (0.547) | 2.967 (0.585) | 2.844 (0.782) | 2.732 (0.624) | 3.094 (0.563) | 2.514 (0.688) | 3.160 (0.686) | 3.569 (0.531) |
| Two or more adults | 3.034 (0.513) | 2.709 (0.616) | 3.290 (0.505) | 2.933 (0.570) | 2.938 (0.723) | 2.794 (0.588) | 2.951 (0.548) | 2.474 (0.673) | 3.038 (0.686) | 3.582 (0.526) |
| Adult(s) and child(ren) | 2.960 (0.491) | 2.534 (0.609) | 3.298 (0.483) | 2.964 (0.553) | 2.831 (0.697) | 2.670 (0.564) | 3.155 (0.505) | 2.504 (0.632) | 3.140 (0.665) | 3.593 (0.521) |
|  |  |  |  |  |  |  |  |  |  |  |
| **Education, R^2^** | .002*** | .002*** | .010*** | .007*** | .002*** | .003*** | .002*** | .035*** | .046*** | .002*** |
| Basic | 3.043 (0.563) | 2.758 (0.650) | 3.322 (0.515) | 2.977 (0.600) | 2.966 (0.738) | 2.845 (0.637) | 2.972 (0.544) | 2.754 (0.650) | 3.325 (0.694) | 3.529 (0.565) |
| Middle | 2.982 (0.535) | 2.620 (0.645) | 3.325 (0.509) | 3.001 (0.570) | 2.861 (0.740) | 2.705 (0.593) | 3.065 (0.539) | 2.602 (0.657) | 3.231 (0.656) | 3.597 (0.517) |
| Academic (lower) | 3.027 (0.495) | 2.659 (0.621) | 3.273 (0.504) | 2.950 (0.560) | 2.906 (0.717) | 2.742 (0.582) | 3.045 (0.547) | 2.443 (0.647) | 3.080 (0.666) | 3.604 (0.518) |
| Academic (upper) | 3.021 (0.498) | 2.639 (0.612) | 3.196 (0.510) | 2.872 (0.561) | 2.855 (0.737) | 2.757 (0.595) | 3.070 (0.554) | 2.332 (0.656) | 2.876 (0.675) | 3.544 (0.544) |
|  |  |  |  |  |  |  |  |  |  |  |
| **HH income, R^2^** | .002*** | .009*** | .001* | .008*** | .001* | .001** | .006*** | .020*** | .103*** | .003*** |
| 1st quartile | 2.979 (0.542) | 2.555 (0.666) | 3.280 (0.526) | 3.010 (0.572) | 2.837 (0.748) | 2.696 (0.606) | 3.105 (0.544) | 2.604 (0.664) | 3.385 (0.621) | 3.602 (0.524) |
| 2nd quartile | 3.024 (0.509) | 2.664 (0.620) | 3.283 (0.512) | 2.979 (0.563) | 2.893 (0.725) | 2.752 (0.593) | 3.076 (0.537) | 2.539 (0.658) | 3.167 (0.639) | 3.600 (0.514) |
| 3rd quartile | 2.978 (0.494) | 2.624 (0.597) | 3.268 (0.492) | 2.909 (0.565) | 2.862 (0.718) | 2.726 (0.567) | 3.034 (0.539) | 2.424 (0.633) | 2.987 (0.645) | 3.557 (0.537) |
| 4th quartile | 3.027 (0.514) | 2.724 (0.612) | 3.247 (0.509) | 2.879 (0.566) | 2.882 (0.737) | 2.746 (0.592) | 2.986 (0.557) | 2.364 (0.656) | 2.780 (0.684) | 3.537 (0.538) |
|  |  |  |  |  |  |  |  |  |  |  |
| **HH special diets, R^2^** | .015*** | .006*** | .002*** | .004*** | .015*** | .019*** | .003*** | .005*** | <.001 | .001** |
| No special diet | 2.969 (0.527) | 2.641 (0.628) | 3.268 (0.508) | 2.918 (0.578) | 2.812 (0.733) | 2.676 (0.597) | 3.038 (0.554) | 2.504 (0.660) | 3.114 (0.683) | 3.562 (0.534) |
| Lactose intolerance | 3.018 (0.493) | 2.705 (0.601) | 3.320 (0.485) | 2.975 (0.556) | 2.906 (0.708) | 2.748 (0.567) | 3.069 (0.529) | 2.533 (0.648) | 3.111 (0.669) | 3.594 (0.520) |
| Gluten-free | 3.133 (0.480) | 2.581 (0.636) | 3.293 (0.520) | 3.018 (0.583) | 3.062 (0.718) | 2.819 (0.579) | 3.033 (0.546) | 2.519 (0.677) | 3.074 (0.683) | 3.612 (0.519) |
| Red-meat-free | 3.181 (0.492) | 2.731 (0.663) | 3.271 (0.524) | 2.997 (0.574) | 3.114 (0.721) | 2.955 (0.606) | 3.023 (0.548) | 2.380 (0.691) | 3.041 (0.701) | 3.609 (0.527) |
| Vegetarians | 3.073 (0.502) | 2.518 (0.656) | 3.220 (0.545) | 3.016 (0.529) | 2.884 (0.778) | 2.915 (0.562) | 3.153 (0.516) | 2.355 (0.654) | 3.077 (0.666) | 3.610 (0.515) |
| Other | 3.046 (0.497) | 2.588 (0.652) | 3.259 (0.527) | 2.981 (0.539) | 2.956 (0.725) | 2.781 (0.585) | 3.090 (0.548) | 2.475 (0.705) | 3.100 (0.708) | 3.620 (0.517) |

HE=health, WC=weight control, SA=sensory appeal, MO=mood control, NC=natural content, EC=ethical concern, CO=convenience, FA=familiarity, PC=price-cheap, PV=price-value, HH=household

^a^R^2^ values represent the proportion of the variance in the absolute food motive that is explained by the given sociodemographic variable.

***P<.001, **P<.01, *P<.05

Additional file 2. Table 2. Results from multiple linear models predicting the absolute importance of health, mood and convenience motive dimensions^a^

|  | Health | | | |  | Mood | | | |  | Convenience | | | |
| --- | --- | --- | --- | --- | --- | --- | --- | --- | --- | --- | --- | --- | --- | --- |
|  | B^b^ | 95% CI | P | ∆R^2c^ |  | B | 95% CI | P | ∆R^2^ |  | B | 95% CI | P | ∆R^2^ |
| **Gender** |  |  |  | 041.*** |  |  |  |  | .034*** |  |  |  |  | .030*** |
| Women | 0.222 | 0.203, 0.242 | <.001 |  |  | 0.226 | 0.205, 0.247 | <.001 |  |  | 0.203 | 0.183, 0.222 | <.001 |  |
| Men |  |  |  |  |  |  |  |  |  |  |  |  |  |  |
| **Age group** |  |  |  | .023*** |  |  |  |  | .013*** |  |  |  |  | .054*** |
| 18-29 |  |  |  |  |  |  |  |  |  |  |  |  |  |  |
| 30-44 | 0.005 | -0.023, 0.034 | .71 |  |  | -0.112 | -0.144, -0.080 | <.001 |  |  | -0.051 | -0.080, -0.021 | .001 |  |
| 45-64 | 0.087 | 0.059, 0.115 | <.001 |  |  | -0.148 | -0.179, -0.118 | <.001 |  |  | -0.224 | -0.253, -0.195 | <.001 |  |
| 65-94 | 0.236 | 0.202, 0.269 | <.001 |  |  | -0.235 | -0.272, -0.198 | <.001 |  |  | -0.378 | -0.412, -0.343 | <.001 |  |
| **Marital status** |  |  |  | <.001 |  |  |  |  | <.001 |  |  |  |  | .005*** |
| Married/cohabiting |  |  |  |  |  |  |  |  |  |  |  |  |  |  |
| Divorced/widowed | 0.008 | -0.019, 0.035 | .56 |  |  | 0.012 | -0.017, 0.042 | .41 |  |  | 0.077 | 0.049, 0.104 | <.001 |  |
| Never married | 0.018 | -0.007, 0.042 | .16 |  |  | 0.016 | -0.011, 0.043 | .25 |  |  | 0.092 | 0.067, 0.117 | <.001 |  |
| **Living situation** |  |  |  | .001** |  |  |  |  | .001*** |  |  |  |  | .008*** |
| Alone | 0.040 | 0.016, 0.065 | .001 |  |  | 0.052 | 0.026, 0.079 | <.001 |  |  | 0.014 | -0.011, 0.039 | .27 |  |
| Two or more adults | 0.034 | 0.012, 0.057 | .003 |  |  | 0.044 | 0.019, 0.068 | .001 |  |  | -0.094 | -0.117, -0.071 | <.001 |  |
| Adult(s) and child(ren) |  |  |  |  |  |  |  |  |  |  |  |  |  |  |
| **Education** |  |  |  | .001*** |  |  |  |  | .007*** |  |  |  |  | .001** |
| Basic | -0.013 | -0.054, 0.028 | .54 |  |  | 0.154 | 0.109, 0.199 | <.001 |  |  | -0.011 | -0.053, 0.031 | .62 |  |
| Middle | -0.040 | -0.064, -0.016 | .001 |  |  | 0.112 | 0.087, 0.138 | <.001 |  |  | -0.028 | -0.052, -0.004 | .023 |  |
| Academic (lower) | 0.003 | -0.021, 0.027 | .81 |  |  | 0.062 | 0.036, 0.089 | <.001 |  |  | -0.046 | -0.071, -0.021 | <.001 |  |
| Academic (upper) |  |  |  |  |  |  |  |  |  |  |  |  |  |  |
| **Household income** |  |  |  | .001* |  |  |  |  | .003*** |  |  |  |  | .001** |
| 1st quartile | -0.034 | -0.061, -0.006 | .017 |  |  | 0.059 | 0.029, 0.089 | <.001 |  |  | 0.009 | -0.019, 0.037 | .54 |  |
| 2nd quartile | -0.014 | -0.040, 0.011 | .26 |  |  | 0.063 | 0.035, 0.091 | <.001 |  |  | 0.043 | 0.018, 0.069 | .001 |  |
| 3rd quartile | -0.039 | -0.068, -0.009 | .010 |  |  | 0.006 | -0.026, 0.038 | .72 |  |  | 0.008 | -0.022, 0.038 | .60 |  |
| 4th quartile |  |  |  |  |  |  |  |  |  |  |  |  |  |  |
| **Special diets in household** |  |  |  | .015*** |  |  |  |  | .002*** |  |  |  |  | .001 |
| No special diet |  |  |  |  |  |  |  |  |  |  |  |  |  |  |
| Lactose-free | 0.053 | 0.030, 0.076 | <.001 |  |  | 0.039 | 0.014, 0.065 | .003 |  |  | 0.008 | -0.015, 0.032 | .49 |  |
| Gluten-free | 0.172 | 0.136, 0.209 | <.001 |  |  | 0.079 | 0.039, 0.120 | <.001 |  |  | -0.034 | -0.072, 0.004 | .08 |  |
| Red-meat-free | 0.209 | 0.167, 0.251 | <.001 |  |  | 0.060 | 0.014, 0.106 | .011 |  |  | -0.035 | -0.079, 0.008 | .11 |  |
| Vegetarians | 0.132 | 0.093, 0.171 | <.001 |  |  | 0.029 | -0.013, 0.072 | .18 |  |  | 0.009 | -0.030, 0.049 | .64 |  |
| Other | 0.084 | 0.046, 0.121 | <.001 |  |  | 0.022 | -0.020, 0.064 | .31 |  |  | -0.007 | -0.046, 0.032 | .74 |  |

^a^Each model includes age, gender and the given sociodemographic variable as predictors.

^b^B values are unstandardized regression coefficients.

^c^∆R^2^ refers to increase in the model R^2^ value after adding the given sociodemographic predictor to the age- and gender-adjusted model.

***P<.001, **P<.01, *P<.05

Additional file 2. Table 3. Results from multiple linear models predicting the absolute importance of sensory appeal, weight control and natural content motive dimensions^a^

|  | Sensory appeal | | | |  | Weight control | | | |  | Natural content | | | |
| --- | --- | --- | --- | --- | --- | --- | --- | --- | --- | --- | --- | --- | --- | --- |
|  | B^b^ | 95% CI | P | ∆R^2c^ |  | B | 95% CI | P | ∆R^2^ |  | B | 95% CI | P | ∆R^2^ |
| **Gender** |  |  |  | .032*** |  |  |  |  | .017*** |  |  |  |  | .037*** |
| Women | 0.195 | 0.176, 0.215 | <.001 |  |  | 0.176 | 0.153, 0.200 | <.001 |  |  | 0.301 | 0.274, 0.328 | <.001 |  |
| Men |  |  |  |  |  |  |  |  |  |  |  |  |  |  |
| **Age group** |  |  |  | .003*** |  |  |  |  | .051*** |  |  |  |  | .050*** |
| 18-29 |  |  |  |  |  |  |  |  |  |  |  |  |  |  |
| 30-44 | 0.019 | -0.010, 0.047 | .20 |  |  | 0.020 | -0.015, 0.055 | .26 |  |  | 0.161 | 0.121, 0.201 | <.001 |  |
| 45-64 | 0.056 | 0.028, 0.084 | <.001 |  |  | 0.260 | 0.226, 0.294 | <.001 |  |  | 0.338 | 0.299, 0.377 | <.001 |  |
| 65-94 | -0.015 | -0.048, 0.018 | .37 |  |  | 0.370 | 0.330, 0.411 | <.001 |  |  | 0.544 | 0.498, 0.591 | <.001 |  |
| **Marital status** |  |  |  | .004*** |  |  |  |  | <.001 |  |  |  |  | .002*** |
| Married/cohabiting |  |  |  |  |  |  |  |  |  |  |  |  |  |  |
| Divorced/widowed | -0.022 | -0.048, 0.005 | .11 |  |  | -0.001 | -0.033, 0.032 | .96 |  |  | -0.046 | -0.083, -0.008 | .02 |  |
| Never married | -0.090 | -0.114, -0.065 | <.001 |  |  | 0.018 | -0.012, 0.048 | .25 |  |  | -0.088 | -0.122, -0.054 | <.001 |  |
| **Living situation** |  |  |  | .003*** |  |  |  |  | .003*** |  |  |  |  | .002*** |
| Alone | -0.062 | -0.087, -0.038 | <.001 |  |  | 0.078 | 0.049, 0.108 | <.001 |  |  | -0.082 | -0.116, -0.048 | <.001 |  |
| Two or more adults | 0.005 | -0.018, 0.027 | .67 |  |  | 0.098 | 0.056, 0.110 | <.001 |  |  | -0.012 | -0.044, 0.019 | .44 |  |
| Adult(s) and child(ren) |  |  |  |  |  |  |  |  |  |  |  |  |  |  |
| **Education** |  |  |  | .009*** |  |  |  |  | .001* |  |  |  |  | .001** |
| Basic | 0.134 | 0.093, 0.175 | <.001 |  |  | 0.038 | -0.011, 0.088 | .13 |  |  | 0.019 | -0.038, 0.076 | .51 |  |
| Middle | 0.122 | 0.099, 0.146 | <.001 |  |  | -0.011 | -0.040, 0.018 | .45 |  |  | 0.012 | -0.021, 0.045 | .48 |  |
| Academic (lower) | 0.069 | 0.045, 0.093 | <.001 |  |  | 0.026 | -0.004, 0.055 | .09 |  |  | 0.056 | 0.022, 0.090 | .001 |  |
| Academic (upper) |  |  |  |  |  |  |  |  |  |  |  |  |  |  |
| **Household income** |  |  |  | <.001 |  |  |  |  | .004*** |  |  |  |  | <.001 |
| 1st quartile | 0.004 | -0.024, 0.031 | .79 |  |  | -0.110 | -0.143, -0.076 | <.001 |  |  | 0.020 | -0.018, 0.058 | .30 |  |
| 2nd quartile | 0.012 | -0.013, 0.038 | .34 |  |  | -0.056 | -0.087, -0.026 | <.001 |  |  | 0.008 | -0.027, 0.043 | .66 |  |
| 3rd quartile | 0.013 | -0.017, 0.042 | .40 |  |  | -0.071 | -0.107, -0.036 | <.001 |  |  | 0.014 | -0.026, 0.055 | .49 |  |
| 4th quartile |  |  |  |  |  |  |  |  |  |  |  |  |  |  |
| **Special diets in household** |  |  |  | .003*** |  |  |  |  | .004*** |  |  |  |  | .016*** |
| No special diet |  |  |  |  |  |  |  |  |  |  |  |  |  |  |
| Lactose-free | 0.041 | 0.018, 0.064 | <.001 |  |  | 0.065 | 0.037, 0.093 | <.001 |  |  | 0.092 | 0.060, 0.124 | <.001 |  |
| Gluten-free | 0.015 | -0.022, 0.051 | .44 |  |  | -0.048 | -0.093, -0.004 | .03 |  |  | 0.260 | 0.209, 0.311 | <.001 |  |
| Red-meat-free | -0.014 | -0.056, 0.028 | .52 |  |  | 0.081 | 0.030, 0.113 | .002 |  |  | 0.285 | 0.226, 0.343 | <.001 |  |
| Vegetarians | -0.076 | -0.115, -0.038 | <.001 |  |  | -0.064 | -0.111, -0.017 | .007 |  |  | 0.139 | 0.085, 0.192 | <.001 |  |
| Other | -0.031 | -0.069, 0.007 | .11 |  |  | -0.033 | -0.079, 0.013 | .16 |  |  | 0.161 | 0.109, 0.214 | <.001 |  |

^a^Each model includes age, gender and the given sociodemographic variable as predictors.

^b^B values are unstandardized regression coefficients.

^c^∆R^2^ refers to increase in the model R^2^ value after adding the given sociodemographic predictor to the age- and gender-adjusted model.

***P<.001, **P<.01, *P<.05

Additional file 2. Table 4. Results from multiple linear models predicting the absolute importance of ethical concern, familiarity and price motive dimensions^a^

|  | Ethical concern | | | |  | Familiarity | | | |  | Price-cheap | | | |  | Price-value | | | |
| --- | --- | --- | --- | --- | --- | --- | --- | --- | --- | --- | --- | --- | --- | --- | --- | --- | --- | --- | --- |
|  | B^b^ | 95% CI | P | ∆R^2c^ |  | B | 95% CI | P | ∆R^2^ |  | B | 95% CI | P | ∆R^2^ |  | B | 95% CI | P | ∆R^2^ |
| **Gender** |  |  |  | .039*** |  |  |  |  | <.001 |  |  |  |  | .001*** |  |  |  |  | .007*** |
| Women | 0.250 | 0.228, 0.272 | <.001 |  |  | -0.013 | -0.038, 0.013 | .33 |  |  | 0.052 | 0.027, 0.078 | <.001 |  |  | 0.093 | 0.073, 0.113 | <.001 |  |
| Men |  |  |  |  |  |  |  |  |  |  |  |  |  |  |  |  |  |  |  |
| **Age group** |  |  |  | .053*** |  |  |  |  | .003*** |  |  |  |  | .014*** |  |  |  |  | .002*** |
| 18-29 |  |  |  |  |  |  |  |  |  |  |  |  |  |  |  |  |  |  |  |
| 30-44 | 0.111 | 0.078, 0.143 | <.001 |  |  | -0.058 | -0.096, -0.020 | .003 |  |  | -0.176 | -0.214, -0.138 | <.001 |  |  | -0.056 | -0.086, -0.026 | <.001 |  |
| 45-64 | 0.252 | 0.221, 0.284 | <.001 |  |  | -0.098 | -0.135, -0.061 | <.001 |  |  | -0.225 | -0.263, -0.188 | <.001 |  |  | -0.048 | -0.077, -0.019 | .001 |  |
| 65-94 | 0.451 | 0.413, 0.489 | <.001 |  |  | -0.025 | -0.069, 0.019 | .27 |  |  | -0.261 | -0.305, -0.216 | <.001 |  |  | -0.085 | -0.119, -0.050 | <.001 |  |
| **Marital status** |  |  |  | <.001* |  |  |  |  | <.001 |  |  |  |  | .009*** |  |  |  |  | <.001 |
| Married/cohabiting |  |  |  |  |  |  |  |  |  |  |  |  |  |  |  |  |  |  |  |
| Divorced/widowed | -0.036 | -0.066, -0.005 | .02 |  |  | -0.004 | -0.039, 0.031 | .82 |  |  | 0.169 | 0.133, 0.204 | <.001 |  |  | 0.011 | -0.016, 0.039 | .42 |  |
| Never married | -0.019 | -0.047, 0.009 | .18 |  |  | 0.038 | 0.005, 0.070 | .02 |  |  | 0.094 | 0.061, 0.127 | <.001 |  |  | -0.022 | -0.047, 0004 | .10 |  |
| **Living situation** |  |  |  | .001** |  |  |  |  | .001* |  |  |  |  | .004*** |  |  |  |  | <.001 |
| Alone | -0.011 | -0.038, 0.017 | .45 |  |  | 0.017 | -0.015, 0.049 | .31 |  |  | 0.061 | 0.029, 0.094 | <.001 |  |  | -0.008 | -0.033, 0.017 | .54 |  |
| Two or more adults | 0.031 | 0.006, 0.057 | .02 |  |  | -0.022 | -0.051, 0.008 | .15 |  |  | -0.042 | -0.072, -0.012 | .006 |  |  | 0.013 | -0.010, 0.037 | .26 |  |
| Adult(s) and child(ren) |  |  |  |  |  |  |  |  |  |  |  |  |  |  |  |  |  |  |  |
| **Education** |  |  |  | .001** |  |  |  |  | .036*** |  |  |  |  | .048*** |  |  |  |  | .002*** |
| Basic | 0.015 | -0.031, 0.062 | .52 |  |  | 0.432 | 0.379, 0.485 | <.001 |  |  | 0.497 | 0.444, 0.551 | <.001 |  |  | <0.001 | -0.043, -0.043 | .99 |  |
| Middle | -0.047 | -0.074, -0.020 | .001 |  |  | 0.269 | 0.239, 0.300 | <.001 |  |  | 0.344 | 0.313, 0.374 | <.001 |  |  | 0.047 | 0.023, 0.072 | <.001 |  |
| Academic (lower) | -0.012 | -0.040, 0.015 | .39 |  |  | 0.111 | 0.079, 0.142 | <.001 |  |  | 0.194 | 0.162, 0.225 | <.001 |  |  | 0.054 | 0.029, 0.079 | <.001 |  |
| Academic (upper) |  |  |  |  |  |  |  |  |  |  |  |  |  |  |  |  |  |  |  |
| **Household income** |  |  |  | <.001 |  |  |  |  | .020*** |  |  |  |  | .092*** |  |  |  |  | .001** |
| 1st quartile | 0.003 | -0.029, 0.034 | .87 |  |  | 0.246 | 0.211, 0.282 | <.001 |  |  | 0.580 | 0.545, 0.615 | <.001 |  |  | 0.039 | 0.011, 0.068 | .007 |  |
| 2nd quartile | 0.001 | -0.027, 0.030 | .93 |  |  | 0.180 | 0.147, 0.213 | <.001 |  |  | 0.379 | 0.346, 0.411 | <.001 |  |  | 0.049 | 0.023, 0.075 | <.001 |  |
| 3rd quartile | 0.007 | -0.026, 0.040 | .69 |  |  | 0.061 | 0.024, 0.099 | .001 |  |  | 0.198 | 0.160, 0.235 | <.001 |  |  | 0.011 | -0.019, 0.041 | .47 |  |
| 4th quartile |  |  |  |  |  |  |  |  |  |  |  |  |  |  |  |  |  |  |  |
| **Special diets in household** |  |  |  | .024*** |  |  |  |  | .006*** |  |  |  |  | .002** |  |  |  |  | .001* |
| No special diet |  |  |  |  |  |  |  |  |  |  |  |  |  |  |  |  |  |  |  |
| Lactose-free | 0.071 | 0.045, 0.097 | <.001 |  |  | 0.028 | -0.003, 0.058 | .08 |  |  | -0.012 | -0.043, 0.019 | .43 |  |  | 0.026 | 0.002, 0.050 | .03 |  |
| Gluten-free | 0.151 | 0.110, 0.192 | <.001 |  |  | 0.013 | -0.035, 0.061 | .60 |  |  | -0.054 | -0.103, -0.005 | .03 |  |  | 0.042 | 0.004, 0.081 | .03 |  |
| Red-meat-free | 0.268 | 0.220, 0.315 | <.001 |  |  | -0.125 | -0.180, -0.069 | <.001 |  |  | -0.079 | -0.135, -0.023 | .006 |  |  | 0.039 | -0.005, 0.083 | .08 |  |
| Vegetarians | 0.295 | 0.252, 0.339 | <.001 |  |  | -0.161 | -0.212, -0.110 | <.001 |  |  | -0.090 | -0.141, -0.038 | .001 |  |  | 0.024 | -0.016, 0.064 | .24 |  |
| Other | 0.121 | 0.078, 0.164 | <.001 |  |  | -0.033 | -0.083, 0.017 | .20 |  |  | -0.040 | -0.091, 0.010 | .12 |  |  | 0.043 | 0.003, 0.083 | .03 |  |

^a^Each model includes age, gender and the given sociodemographic variable as predictors.

^b^B values are unstandardized regression coefficients.

^c^∆R^2^ refers to increase in the model R^2^ value after adding the given sociodemographic predictor to the age- and gender-adjusted model.

***P<.001, **P<.01, *P<.05
