# Supplementary material for: Sociodemographic differences in motives for food selection: results from the LoCard cross-sectional survey
Source: Int J Behav Nutr Phys Act. 2021 Jun 2;18:71. doi: 10.1186/s12966-021-01139-2 (PMC8173871; doi:10.1186/s12966-021-01139-2)
Supplement: Supplementary file 3 — Additional file 3: Table 1. Results from re-weighted multiple linear models predicting the relative importance of health, mood and convenience motive dimensions. Table 2. Results from re-weighted multiple linear models predicting the relative importance of sensory appeal, weight control and natural content motive dimensions. Table 3. Results from re-weighted multiple linear models predicting the relative importance of ethical concern, familiarity and price motive dimensions. [file 12966_2021_1139_MOESM3_ESM.docx]

Additional file 3. Table 1. Results from re-weighted multiple linear models predicting the relative importance of health, mood and convenience motives^a,b^

|  | Health | | | |  | Mood | | | |  | Convenience | | | |
| --- | --- | --- | --- | --- | --- | --- | --- | --- | --- | --- | --- | --- | --- | --- |
|  | B^c^ | 95% CI | P | ∆R^2d^ |  | B | 95% CI | P | ∆R^2^ |  | B | 95% CI | P | ∆R^2^ |
| **Gender** |  |  |  | .004*** |  |  |  |  | <.001 |  |  |  |  | <.001 |
| Women | 0.014 | 0.009, 0.018 | <.001 |  |  | 0.003 | -0.002, 0.009 | .26 |  |  | 0.002 | -0.005, 0.009 | .57 |  |
| Men |  |  |  |  |  |  |  |  |  |  |  |  |  |  |
| **Age group** |  |  |  | .015*** |  |  |  |  | .067*** |  |  |  |  | .107*** |
| 17-29 |  |  |  |  |  |  |  |  |  |  |  |  |  |  |
| 30-44 | -0.003 | -0.009, 0.004 | .39 |  |  | -0.040 | -0.049, -0.032 | <.001 |  |  | -0.025 | -0.036, -0.014 | <.001 |  |
| 45-64 | 0.006 | <0.001, 0.013 | .042 |  |  | -0.076 | -0.085, -0.068 | <.001 |  |  | -0.102 | -0.112, -0.091 | <.001 |  |
| 65-94 | 0.034 | 0.027, 0.040 | <.001 |  |  | -0.117 | -0.126, -0.108 | <.001 |  |  | -0.172 | -0.182, -0.161 | <.001 |  |
| **Marital status** |  |  |  | <.001* |  |  |  |  | <.001** |  |  |  |  | .008*** |
| Married/cohabiting |  |  |  |  |  |  |  |  |  |  |  |  |  |  |
| Divorced/widowed | 0.005 | -0.001, 0.011 | .12 |  |  | 0.004 | -0.004, 0.012 | .34 |  |  | 0.028 | 0.019, 0.038 | <.001 |  |
| Never married | 0.007 | <0.001, 0.013 | .039 |  |  | 0.013 | 0.005, 0.022 | .002 |  |  | 0.043 | 0.033, 0.053 | <.001 |  |
| **Living situation** |  |  |  | .002*** |  |  |  |  | .002*** |  |  |  |  | .008*** |
| Alone | 0.012 | 0.006, 0.017 | <.001 |  |  | 0.018 | 0.011, 0.026 | <.001 |  |  | 0.007 | -0.002, 0.016 | .14 |  |
| Two or more adults | 0.008 | 0.002, 0.013 | .006 |  |  | 0.009 | 0.002, 0.016 | .017 |  |  | -0.035 | -0.044, -0.026 | <.001 |  |
| Adult(s) and child(ren) |  |  |  |  |  |  |  |  |  |  |  |  |  |  |
| **Education** |  |  |  | .009*** |  |  |  |  | .004*** |  |  |  |  | .003*** |
| Basic | -0.037 | -0.045, -0.029 | <.001 |  |  | 0.029 | 0.018, 0.040 | <.001 |  |  | -0.037 | -0.051, -0.024 | <.001 |  |
| Middle | -0.024 | -0.032, -0.017 | <.001 |  |  | 0.028 | 0.018, 0.037 | <.001 |  |  | -0.029 | -0.041, -0.017 | <.001 |  |
| Academic (lower) | -0.013 | -0.021, -0.005 | .001 |  |  | 0.013 | 0.003, 0.024 | .014 |  |  | -0.031 | -0.044, -0.018 | <.001 |  |
| Academic (upper) |  |  |  |  |  |  |  |  |  |  |  |  |  |  |
| **Household income** |  |  |  | .005*** |  |  |  |  | .003*** |  |  |  |  | <.001 |
| 1st quartile | -0.024 | -0.031, -0.017 | <.001 |  |  | 0.022 | 0.013, 0.031 | <.001 |  |  | -0.004 | -0.015, 0.008 | .55 |  |
| 2nd quartile | -0.012 | -0.019, -0.005 | <.001 |  |  | 0.014 | 0.005, 0.023 | .002 |  |  | 0.002 | -0.009, 0.013 | .75 |  |
| 3rd quartile | -0.018 | -0.026, -0.010 | <.001 |  |  | -0.001 | -0.011, 0.009 | .85 |  |  | 0.010 | -0.003, 0.023 | .12 |  |
| 4th quartile |  |  |  |  |  |  |  |  |  |  |  |  |  |  |
| **Special diets in household** |  |  |  | .013*** |  |  |  |  | .002** |  |  |  |  | .005*** |
| No special diet |  |  |  |  |  |  |  |  |  |  |  |  |  |  |
| Lactose-free | -0.006 | -0.011, <0.001 | .037 |  |  | -0.006 | -0.013, 0.002 | .13 |  |  | -0.012 | -0.021, -0.003 | .008 |  |
| Gluten-free | 0.037 | 0.028, 0.046 | <.001 |  |  | -0.003 | -0.015, 0.009 | .61 |  |  | -0.040 | -0.055, -0.025 | <.001 |  |
| No red meat | 0.035 | 0.024, 0.047 | <.001 |  |  | -0.027 | -0.042, -0.012 | <.001 |  |  | -0.044 | -0.062, -0.025 | <.001 |  |
| Vegetarians | 0.023 | 0.013, 0.033 | <.001 |  |  | -0.013 | -0.026, 0.001 | .063 |  |  | -0.029 | -0.045, -0.012 | .001 |  |
| Other | 0.019 | 0.009, 0.028 | <.001 |  |  | -0.018 | -0.031, -0.005 | .007 |  |  | -0.020 | -0.036, -0.003 | .018 |  |

^a^Using post-stratification weights developed to correct the sociodemographic distribution of the LoCard participants closer to the Finnish adult population [26].

^b^Each regression model includes age, gender and the given sociodemographic variable as predictors.

^c^B values are unstandardized regression coefficients.

^d^∆R^2^ refers to increase in the model R^2^ value after adding the given sociodemographic predictor to the age- and gender-adjusted model.

***P<.001, **P<.01, *P<.05

Additional file 3. Table 2. Results from re-weighted multiple linear models predicting the relative importance of sensory appeal, weight control and natural content motives^a,b^

|  | Sensory appeal | | | |  | Weight control | | | |  | Natural content | | | |
| --- | --- | --- | --- | --- | --- | --- | --- | --- | --- | --- | --- | --- | --- | --- |
|  | B^c^ | 95% CI | P | ∆R^2d^ |  | B | 95% CI | P | ∆R^2^ |  | B | 95% CI | P | ∆R^2^ |
| **Gender** |  |  |  | .001*** |  |  |  |  | <.001** |  |  |  |  | .006*** |
| Women | -0.011 | -0.018, -0.005 | <.001 |  |  | 0.011 | 0.004, 0.018 | .002 |  |  | 0.033 | 0.025, 0.041 | <.001 |  |
| Men |  |  |  |  |  |  |  |  |  |  |  |  |  |  |
| **Age group** |  |  |  | .023*** |  |  |  |  | .053*** |  |  |  |  | .053*** |
| 17-29 |  |  |  |  |  |  |  |  |  |  |  |  |  |  |
| 30-44 | 0.008 | -0.001, 0.018 | .09 |  |  | 0.020 | 0.010, 0.030 | <.001 |  |  | 0.046 | 0.034, 0.057 | <.001 |  |
| 45-64 | -0.005 | -0.014, 0.004 | .31 |  |  | 0.087 | 0.077, 0.097 | <.001 |  |  | 0.091 | 0.080, 0.102 | <.001 |  |
| 65-94 | -0.058 | -0.068, -0.048 | <.001 |  |  | 0.103 | 0.093, 0.114 | <.001 |  |  | 0.141 | 0.129, 0.153 | <.001 |  |
| **Marital status** |  |  |  | .006*** |  |  |  |  | <.001** |  |  |  |  | .005*** |
| Married/cohabiting |  |  |  |  |  |  |  |  |  |  |  |  |  |  |
| Divorced/widowed | -0.010 | -0.019, -0.002 | .018 |  |  | -0.016 | -0.025, -0.006 | .001 |  |  | -0.021 | -0.032, -0.010 | <.001 |  |
| Never married | -0.036 | -0.045, -0.027 | <.001 |  |  | -0.004 | -0.014, 0.006 | .48 |  |  | -0.042 | -0.053, -0.030 | <.001 |  |
| **Living situation** |  |  |  | .005*** |  |  |  |  | .004*** |  |  |  |  | .004*** |
| Alone | -0.030 | -0.038, -0.021 | <.001 |  |  | 0.013 | 0.004, 0.022 | .005 |  |  | -0.031 | -0.042, -0.021 | <.001 |  |
| Two or more adults | -0.009 | -0.017, -0.001 | .024 |  |  | 0.028 | 0.019, 0.036 | <.001 |  |  | -0.004 | -0.014, 0.005 | .38 |  |
| Adult(s) and child(ren) |  |  |  |  |  |  |  |  |  |  |  |  |  |  |
| **Education** |  |  |  | .002*** |  |  |  |  | .004*** |  |  |  |  | .003*** |
| Basic | 0.022 | 0.011, 0.034 | <.001 |  |  | -0.023 | -0.036, -0.011 | <.001 |  |  | -0.023 | -0.037, -0.008 | .002 |  |
| Middle | 0.024 | 0.014, 0.035 | <.001 |  |  | -0.025 | -0.037, -0.014 | <.001 |  |  | -0.005 | -0.018, 0.008 | .47 |  |
| Academic (lower) | 0.016 | 0.005, 0.028 | .006 |  |  | 0.001 | -0.012, 0.013 | .91 |  |  | 0.010 | -0.004, 0.025 | .15 |  |
| Academic (upper) |  |  |  |  |  |  |  |  |  |  |  |  |  |  |
| **Household income** |  |  |  | .001* |  |  |  |  | .010*** |  |  |  |  | <.001 |
| 1st quartile | -0.005 | -0.015, 0.005 | .32 |  |  | -0.057 | -0.068, -0.046 | <.001 |  |  | -0.010 | -0.022, 0.003 | .13 |  |
| 2nd quartile | -0.008 | -0.018, 0.001 | .10 |  |  | -0.038 | -0.049, -0.027 | <.001 |  |  | -0.002 | -0.015, 0.010 | .71 |  |
| 3rd quartile | 0.007 | -0.004, 0.019 | .22 |  |  | -0.029 | -0.041, -0.017 | <.001 |  |  | 0.003 | -0.011, 0.018 | .65 |  |
| 4th quartile |  |  |  |  |  |  |  |  |  |  |  |  |  |  |
| **Special diets in household** |  |  |  | .011*** |  |  |  |  | .004*** |  |  |  |  | .014*** |
| No special diet |  |  |  |  |  |  |  |  |  |  |  |  |  |  |
| Lactose-free | -0.001 | -0.009, 0.007 | .76 |  |  | -0.002 | -0.010, 0.007 | .69 |  |  | 0.024 | 0.015, 0.034 | <.001 |  |
| Gluten-free | -0.039 | -0.052, -0.026 | <.001 |  |  | -0.035 | -0.049, -0.021 | <.001 |  |  | 0.082 | 0.066, 0.098 | <.001 |  |
| No red meat | -0.026 | -0.043, -0.010 | .001 |  |  | -0.002 | -0.020, 0.015 | .81 |  |  | 0.061 | 0.041, 0.082 | <.001 |  |
| Vegetarians | -0.059 | -0.073, -0.044 | <.001 |  |  | -0.035 | -0.051, -0.019 | <.001 |  |  | 0.039 | 0.021, 0.057 | <.001 |  |
| Other | -0.034 | -0.048, -0.019 | <.001 |  |  | -0.027 | -0.043, -0.012 | .001 |  |  | 0.060 | 0.042, 0.078 | <.001 |  |

^a^Using post-stratification weights developed to correct the sociodemographic distribution of the LoCard participants closer to the Finnish adult population [26].

^b^Each regression model includes age, gender and the given sociodemographic variable as predictors.

^c^B values are unstandardized regression coefficients.

^d^∆R^2^ refers to increase in the model R^2^ value after adding the given sociodemographic predictor to the age- and gender-adjusted model.

***P<.001, **P<.01, *P<.05

Additional file 3. Table 3. Results from re-weighted multiple linear models predicting the relative importance of ethical concern, familiarity and price motives^a,b^

|  | Ethical concern | | | |  | Familiarity | | | |  | Price, cheap | | | |  | Price, value | | | |
| --- | --- | --- | --- | --- | --- | --- | --- | --- | --- | --- | --- | --- | --- | --- | --- | --- | --- | --- | --- |
|  | B^c^ | 95% CI | P | ∆R^2d^ |  | B | 95% CI | P | ∆R^2^ |  | B | 95% CI | P | ∆R^2^ |  | B | 95% CI | P | ∆R^2^ |
| **Gender** |  |  |  | .005*** |  |  |  |  | .021*** |  |  |  |  | .019*** |  |  |  |  | .020*** |
| Women | 0.021 | 0.016, 0.027 | <.001 |  |  | -0.063 | -0.071, -0.054 | <.001 |  |  | -0.070 | -0.079, -0.061 | <.001 |  |  | -0.058 | -0.066, -0.051 | <.001 |  |
| Men |  |  |  |  |  |  |  |  |  |  |  |  |  |  |  |  |  |  |  |
| **Age group** |  |  |  | .062*** |  |  |  |  | .006*** |  |  |  |  | .027*** |  |  |  |  | .029*** |
| 17-29 |  |  |  |  |  |  |  |  |  |  |  |  |  |  |  |  |  |  |  |
| 30-44 | 0.032 | 0.023, 0.040 | <.001 |  |  | -0.027 | -0.039, -0.015 | <.001 |  |  | -0.047 | -0.061, -0.033 | <.001 |  |  | -0.024 | -0.035, -0.012 | <.001 |  |
| 45-64 | 0.061 | 0.053, 0.069 | <.001 |  |  | -0.049 | -0.061, -0.037 | <.001 |  |  | -0.083 | -0.097, -0.070 | <.001 |  |  | -0.050 | -0.061, -0.039 | <.001 |  |
| 65-94 | 0.110 | 0.102, 0.119 | <.001 |  |  | -0.026 | -0.038, -0.013 | <.001 |  |  | -0.121 | -0.136, -0.107 | <.001 |  |  | -0.100 | -0.112, -0.088 | <.001 |  |
| **Marital status** |  |  |  | .002*** |  |  |  |  | <.001* |  |  |  |  | .009*** |  |  |  |  | <.001 |
| Married/cohabiting |  |  |  |  |  |  |  |  |  |  |  |  |  |  |  |  |  |  |  |
| Divorced/widowed | -0.012 | -0.020, -0.004 | .002 |  |  | 0.004 | -0.007, 0.016 | .42 |  |  | 0.056 | 0.043, 0.069 | <.001 |  |  | -0.003 | -0.014, 0.007 | .55 |  |
| Never married | -0.015 | -0.023, -0.006 | .001 |  |  | 0.019 | 0.006, 0.031 | .003 |  |  | 0.041 | 0.027, 0.055 | <.001 |  |  | -0.005 | -0.016, 0007 | .43 |  |
| **Living situation** |  |  |  | .002*** |  |  |  |  | <.001 |  |  |  |  | .008*** |  |  |  |  | <.001 |
| Alone | -0.008 | -0.015, -0.001 | .041 |  |  | -0.001 | -0.011, 0.011 | .95 |  |  | 0.019 | 0.007, 0.032 | .003 |  |  | -0.001 | -0.012, 0.009 | .79 |  |
| Two or more adults | 0.012 | 0.005, 0.019 | .001 |  |  | -0.011 | -0.021, -0.001 | .042 |  |  | -0.037 | -0.049, -0.025 | <.001 |  |  | -0.006 | -0.016, 0.003 | .21 |  |
| Adult(s) and child(ren) |  |  |  |  |  |  |  |  |  |  |  |  |  |  |  |  |  |  |  |
| **Education** |  |  |  | .003*** |  |  |  |  | .033*** |  |  |  |  | .028*** |  |  |  |  | .005*** |
| Basic | -0.019 | -0.029, -0.008 | <.001 |  |  | 0.121 | 0.106, 0.136 | <.001 |  |  | 0.145 | 0.127, 0.162 | <.001 |  |  | -0.032 | -0.046, -0.018 | <.001 |  |
| Middle | -0.024 | -0.034, -0.015 | <.001 |  |  | 0.079 | 0.066, 0.093 | <.001 |  |  | 0.103 | 0.088, 0.119 | <.001 |  |  | -0.001 | -0.014, 0.012 | .89 |  |
| Academic (lower) | -0.014 | -0.024, -0.003 | .009 |  |  | 0.025 | 0.010, 0.040 | .001 |  |  | 0.060 | 0.043, 0.077 | <.001 |  |  | 0.006 | -0.008, 0.020 | .38 |  |
| Academic (upper) |  |  |  |  |  |  |  |  |  |  |  |  |  |  |  |  |  |  |  |
| **Household income** |  |  |  | .002*** |  |  |  |  | .014*** |  |  |  |  | .058*** |  |  |  |  | <.001 |
| 1st quartile | -0.009 | -0.018, -0.001 | .044 |  |  | 0.071 | 0.058, 0.084 | <.001 |  |  | 0.179 | 0.164, 0.194 | <.001 |  |  | -0.003 | -0.015, 0.010 | .68 |  |
| 2nd quartile | -0.011 | -0.020, -0.002 | .015 |  |  | 0.046 | 0.034, 0.059 | <.001 |  |  | 0.116 | 0.102, 0.131 | <.001 |  |  | 0.001 | -0.011, 0.013 | .92 |  |
| 3rd quartile | 0.006 | -0.004, 0.016 | .26 |  |  | 0.014 | -0.001, 0.029 | .059 |  |  | 0.060 | 0.043, 0.076 | <.001 |  |  | 0.005 | -0.009, 0.019 | .51 |  |
| 4th quartile |  |  |  |  |  |  |  |  |  |  |  |  |  |  |  |  |  |  |  |
| **Special diets in household** |  |  |  | .020*** |  |  |  |  | .009*** |  |  |  |  | .010*** |  |  |  |  | .003*** |
| No special diet |  |  |  |  |  |  |  |  |  |  |  |  |  |  |  |  |  |  |  |
| Lactose-free | 0.016 | 0.009, 0.023 | <.001 |  |  | -0.001 | -0.011, 0.010 | .97 |  |  | -0.033 | -0.045, -0.021 | <.001 |  |  | -0.005 | -0.015, 0.005 | .32 |  |
| Gluten-free | 0.024 | 0.012, 0.035 | <.001 |  |  | -0.016 | -0.033, 0.001 | .064 |  |  | -0.055 | -0.074, -0.035 | <.001 |  |  | -0.030 | -0.046, -0.014 | <.001 |  |
| No red meat | 0.056 | 0.042, 0.070 | <.001 |  |  | -0.076 | -0.098, -0.055 | <.001 |  |  | -0.089 | -0.114, -0.065 | <.001 |  |  | -0.041 | -0.061, -0.021 | <.001 |  |
| Vegetarians | 0.084 | 0.071, 0.097 | <.001 |  |  | -0.065 | -0.084, -0.046 | <.001 |  |  | -0.051 | -0.074, -0.029 | <.001 |  |  | -0.029 | -0.047, -0.011 | .002 |  |
| Other | 0.037 | 0.024, 0.050 | <.001 |  |  | -0.022 | -0.041, -0.003 | .023 |  |  | -0.053 | -0.075, -0.031 | <.001 |  |  | -0.006 | -0.024, 0.012 | .51 |  |

^a^Using post-stratification weights developed to correct the sociodemographic distribution of the LoCard participants closer to the Finnish adult population [26].

^b^Each regression model includes age, gender and the given sociodemographic variable as predictors.

^c^B values are unstandardized regression coefficients.

^d^∆R^2^ refers to increase in the model R^2^ value after adding the given sociodemographic predictor to the age- and gender-adjusted model.

***P<.001, **P<.01, *P<.05
